# Electron competition in azobenzene programs infection-adaptive antibacterial therapy

**DOI:** 10.1126/sciadv.aee7761

**Published:** 2026-11-06

**Authors:** Yongjin Hu, Jiabo Li, Guixian Wang, Xiao Wang, Zhiyuan Zhu, Jingyi Rao

**Affiliations:** ^1^ Department of Urology, Tongji Hospital, Tongji Medical College, Huazhong University of Science and Technology, Wuhan, Hubei 430074, P. R. China.; ^2^ State Key Laboratory of Materials Processing and Die & Mould Technology, and Hubei Key Laboratory of Bioinorganic Chemistry and Materia Medica, School of Chemistry and Chemical Engineering, Huazhong University of Science and Technology, Wuhan 430074, P. R. China.; ^3^ Taizhou Research Institute, Southern University of Science and Technology, Taizhou, Zhejiang, 318001, P. R. China.

## Abstract

Conventional stimulus-responsive antibacterial systems generally operate through binary trigger–activation mechanisms, making them poorly suited to evolving infection microenvironments and thereby contributing to persistent infection and resistance development. Here, we establish an azobenzene-based kinetic regulatory system in which oxygen gradients commonly present in infected tissues continuously program reaction kinetics through electron competition. Oxygen acts as a competing electron acceptor within the reductive reaction network, partitioning electron transfer between azo reduction and oxygen consumption. Substituent electronic structures further reshape reduction propensity, encoding distinct kinetic behaviors. These electronically programmed kinetics translated into adaptive nanoscale dissociation and drug-release profiles: Electron-rich azobenzenes rapidly disassembled under severe hypoxia, whereas electron-withdrawing structures sustained release under mild hypoxia, generating infection state–adaptive antibacterial outputs. Moreover, the same electronic features governing reduction kinetics also reduced the reactivity and toxicity of reductive metabolites. This work establishes electron competition as a strategy for converting environmental gradients into continuous kinetic outputs for adaptive antibacterial therapy.

## INTRODUCTION

Bacterial infection is not a static process, but a continuously evolving microenvironment shaped by bacterial burden, tissue metabolism, and oxygen (O_2_) consumption (*1*). Different infection stages therefore impose distinct antibacterial kinetic demands. Regions with high bacterial loads require rapid and potent bactericidal activity, whereas low-burden regions benefit more from mild yet sustained therapeutic maintenance to minimize resistance selection and off-target toxicity (*2*–*4*). Ideally, antibacterial materials should dynamically adjust their reaction rates and therapeutic intensity in response to pathological evolution, thereby matching antibacterial kinetics with disease progression (*4*–*8*). However, most stimulus-responsive systems still operate through a conventional “trigger-response” paradigm, in which environmental cues merely determine whether a system is activated rather than regulating the reaction process itself (*9*–*11*). As a result, their outputs are typically limited to binary transition between inactive and fully activated states, making continuous kinetic adaptation difficult (*12*, *13*). Overcoming this limitation requires reaction mechanisms in which environmental variables directly regulate reaction kinetics.

Azobenzene bioreduction provides a representative framework for establishing this kinetically regulated behavior. In this process, electrons transferred during enzymatic reduction can either drive azo-bond cleavage or be intercepted by O_2_ (*14*). Because O_2_ gradients are ubiquitous in infected tissues and bacterial biofilms, O_2_ can continuously influence reduction kinetics by competing for electrons within the reaction network (*7*, *15*–*17*). Under these conditions, O_2_ no longer functions merely as an external trigger for activation but instead acts as a kinetic regulator that reshapes reaction rates across different hypoxic environments. Meanwhile, substituent electronic structures further modulate the electron-accepting propensity of azobenzene, thereby altering cleavage kinetics and reduction pathways (*16*, *18*). Consequently, environmental O_2_ levels and molecular electronic structures cooperatively define the kinetic landscape of azobenzene bioreduction: O_2_ determines the surrounding reaction environment, whereas substituent electronics encode intrinsic reduction behavior. This mechanism moves azobenzene systems beyond conventional hypoxia-triggered responses toward continuously programmable kinetic regulation.

For biomedical applications, however, controlled kinetics alone is insufficient unless accompanied by predictable biological consequences. The biomedical translation of azobenzene has long been constrained by the potential toxicity of its reductive metabolites (*19*). Aromatic amine products generated from azo-bond cleavage may induce oxidative stress, metabolic activation, and cellular damage, ultimately causing organ toxicity and genotoxicity (*20*–*22*). Mechanistically, the chemical reactivity of these metabolites is likewise strongly influenced by substituent electronic structures. Thus, the same molecular electronic features governing azobenzene reduction kinetics may also determine the biological consequences of the resulting degradation products. This coupling creates an opportunity to synchronously regulate therapeutic behavior and biosafety through unified electronic-structure design.

Here, we establish an azobenzene-based kinetic regulatory platform in which O_2_ participates directly in reaction network–controlled electron competition (Fig. 1). Within this framework, O_2_ gradients continuously regulate reduction kinetics, while substituent electronics programmably reshape kinetic sensitivity and reaction behavior. These electronically encoded kinetic differences are further translated across scales into nanoscale structural evolution and adaptive drug-release profiles, enabling therapeutic outputs that dynamically shift from rapid bactericidal release to sustained maintenance release under distinct hypoxic conditions. At the same time, electronic regulation together with nanoscale confinement reduces the reactivity of reductive metabolites, thereby improving biosafety alongside therapeutic performance. Collectively, this work demonstrates that O_2_-participated reaction networks can directly convert environmental gradients into programmable therapeutic kinetics, providing a chemical framework for adaptive antibacterial materials in complex physiological environments.

**Fig. 1.**
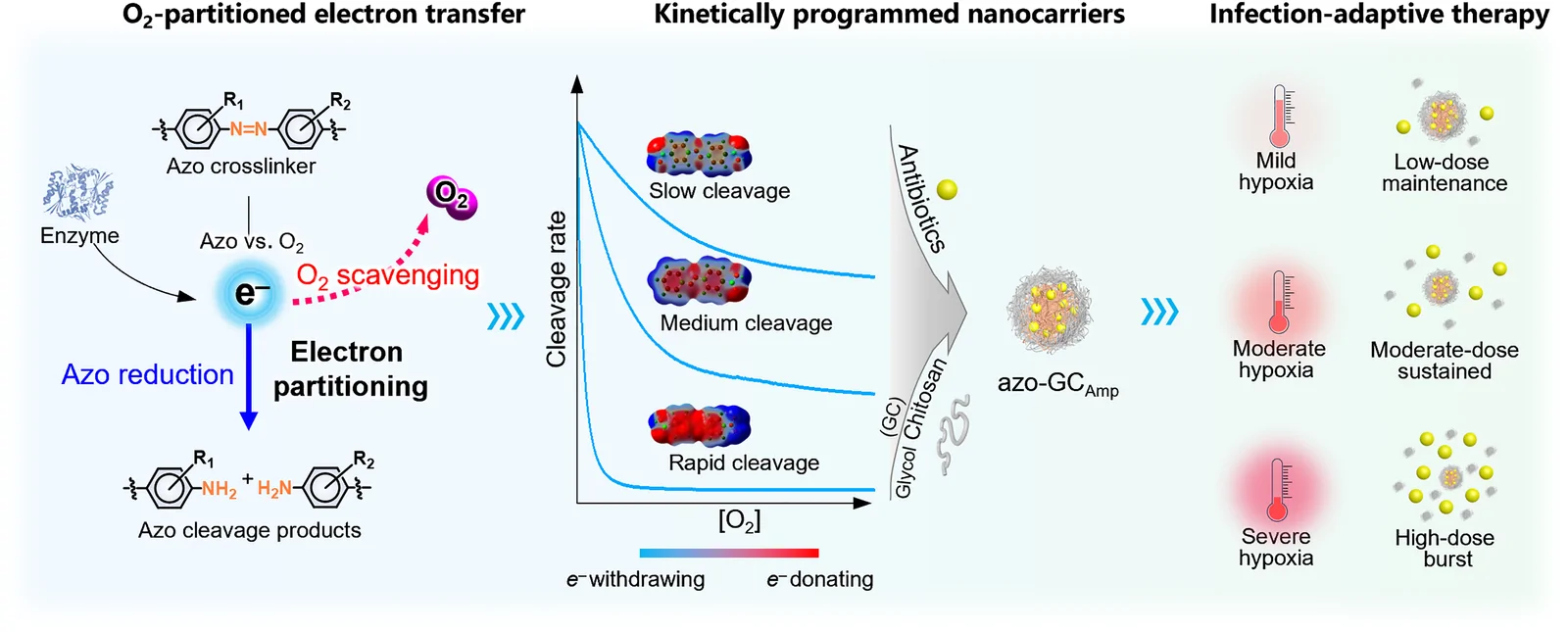
Conceptual framework of electron-competition-controlled kinetic programming for infection-adaptive antibacterial therapy.

## RESULTS

### Oxygen-partitioned electron transfer governs azobenzene bioreduction kinetics

In physiological conditions, azobenzene bioreduction proceeds through a sequential two-electron transfer process mediated by bacterial azoR and nicotinamide adenine dinucleotide phosphate (NADPH)/riboflavin 5′-phosphate (FMN) cofactor system (Fig. 2A). (*14*, *23*, *24*) NADPH first transfers a hydride ion to FMN, producing the reduced intermediate FMNH^−^. FMNH^−^ then donates an electron to the azo (─N═N─) bond, forming an azo^−^ intermediate, which undergoes protonation to form azoH^•^. FMN^−^ then transfers a second electron to azoH^•^, generating azoH^−^, followed by protonation-induced N─N cleavage and the formation of two aniline derivatives. Thus, productive azobenzene cleavage depends on continuous electron flux through the FMN-mediated reduction pathway.

**Fig. 2.**
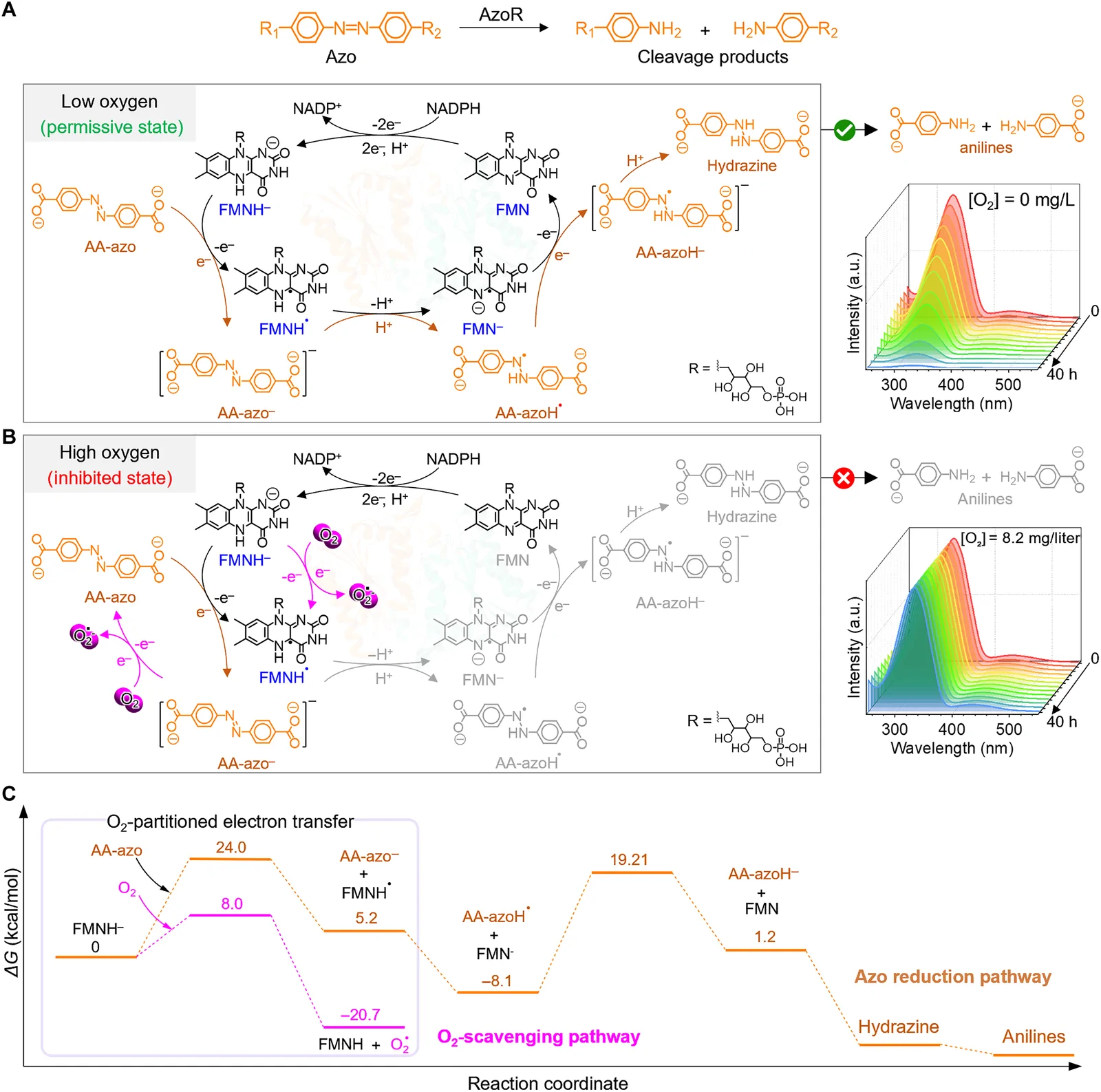
O_2_-partitioned electron transfer regulates azobenzene bioreduction kinetics through competing reduction pathways. (**A**) Under low-O_2_ conditions (permissive state), electron flux preferentially proceeds through the azo reduction pathway, resulting in azo-bond cleavage and formation of aniline products. (**B**) Under high-O_2_ conditions (inhibited state), O_2_ competes for electrons flux through the O_2_-scavenging pathway, thereby suppressing productive azo reduction and cleavage. (**C**) DFT-calculated free energy profiles of the competing azo reduction and O_2_-scavenging pathways originating from reduced flavin intermediates. a.u., arbitrary unit.

O_2_ directly participates in this reaction network as a competing electron acceptor rather than merely serving as an environmental trigger (Fig. 2B). Density functional theory (DFT) calculations showed that the activation energy barrier (Δ*G*^†^) for electron transfer from FMNH^−^ to O_2_ is only 8.0 kcal/mol, much lower than that for transfer to azobenzene (Fig. 2C). This energetic preference enables O_2_ to intercept electron flow before productive azo reduction occurs. Specifically, O_2_ competes with azobenzene at two levels: It preferentially captures electrons from FMNH^−^ to generate superoxide, thereby acting as an electron-scavenging pathway, and rapidly oxidizes the azo- intermediate back to the parent azo state. As a result, the overall reduction outcome is determined by electron partitioning between O_2_-scavenging and azo-bond reduction pathways.

Our results reveal that O_2_ acts as a kinetic gate for productive azo reduction. Under low-O_2_ conditions, electron scavenging by O_2_ is minimized, allowing electron flux to preferentially proceed toward azo reduction and establishing a permissive state for bond cleavage (Fig. 2A). In contrast, under high O_2_ levels, O_2_ intercepts electron flow and suppresses productive reduction, generating an inhibited state (Fig. 2B). This permissive-to-inhibited transition functionally resembles an O_2_-controlled gating process.

To experimentally validate this O_2_-controlled electron competition mechanism, we synthesized **AA-azo**, a systematic azobenzene derivative bearing para-carboxyl substituents (Fig. 3A and fig. S1). The electron-withdrawing **AA**-type structure provides a well-defined model for probing O_2_-regulated electron transfer while simultaneously enabling nanocarrier crosslinking. The structure of **AA-azo** was confirmed by proton nuclear magnetic resonance (^1^H-NMR) spectroscopy (fig. S2). Under severe hypoxia [O_2_ (<0.1 mg/liter)] with azoR and NADPH, the π-π* absorption band of **AA-azo** at ∼340 nm rapidly decreased, indicating efficient azo cleavage (Fig. 2A and fig. S3). In contrast, under normoxia [O_2_ (8 mg/liter)], the absorption remained nearly unchanged, confirming that O_2_ scavenging effectively suppresses productive azo reduction (Fig. 2B). DFT calculations further supported this O_2_-competitive electron-transfer mechanism (Fig. 2C). These results experimentally support an O_2_-controlled kinetic gating mechanism.

**Fig. 3.**
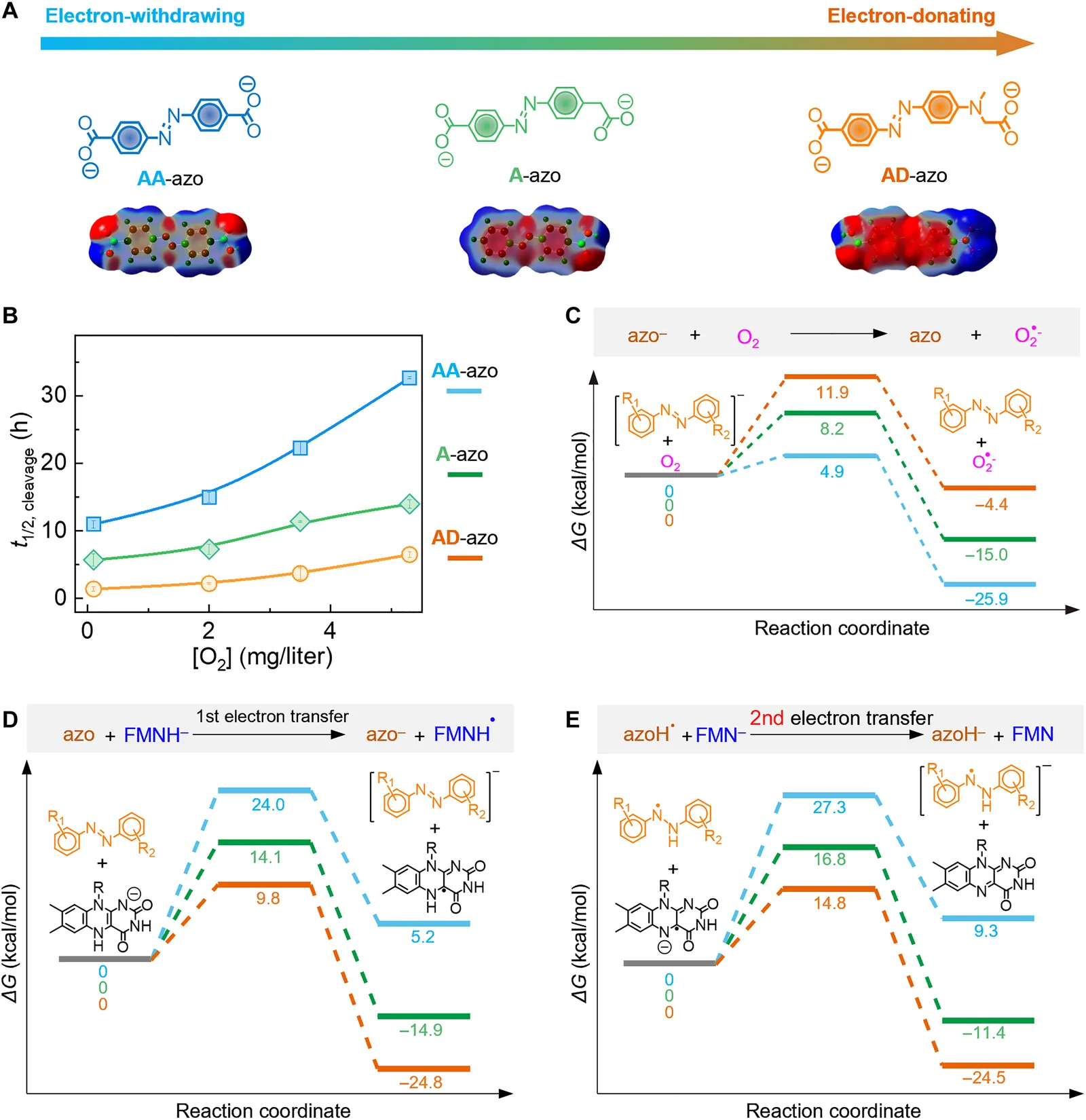
Electronic substituent effects regulate electron partitioning between O_2_ scavenging and azo reduction pathways. (**A**) Chemical structures and electrostatic potential surface maps of azo-based crosslinkers. (**B**) Cleavage half-lives (*t*_1/2_) under different O_2_ concentrations. Data are presented as means ± SD (*n* = 3). (**C** to **E**) DFT-calculated free energy profiles of (C) the O_2_-scavenging pathway and (D and E) the first and second electron-transfer steps in the azo reduction pathway.

To further quantify how O_2_ regulated reduction kinetics, we monitored **AA-azo** cleavage under different O_2_ concentrations. Under severe hypoxia [O_2_ (<0.1 mg/liter)], the π-π* absorption peak of **AA-azo** at ∼340 nm decreased continuously, yielding a half-life (*t*_1/2_) of 10.7 hours and indicating effective electron transfer toward azo reduction (Fig. 3B). Increasing O_2_ to mild hypoxia [O_2_ (2 to 5.3 mg/liter)] progressively slowed the reduction process, extending *t*_1/2_ to 32.5 hours. Under normoxia [O_2_ (8 mg/liter)], electron transfer toward the azo bond was almost completely blocked, and cleavage efficiency fell below 5% (Fig. 2B).

This graded behavior can be attributed to O_2_-dependent redistribution of electron flux within the competing reaction network. As O_2_ concentration increases, progressively more electrons are diverted into the O_2_-scavenging pathway rather than azo-bond reduction, slowing and eventually suppressing cleavage. The O_2_-sensitive azo^−^ intermediate further amplifies this inhibition through oxidative quenching (Fig. 2, B and C). Therefore, O_2_ not only determines whether reduction is permissible but also continuously tunes the magnitude and sensitivity of the kinetic output. This O_2_-partitioned electron-transfer framework converts continuous O_2_ gradients into graded kinetic outputs, enabling tunable modulation of reaction rate and O_2_ sensitivity.

### Electronic substituent effects encode programmable bioreduction kinetics

Having established that O_2_ regulates reaction permissibility through electron competition, we next investigated whether the competition kinetics themselves could be programmably tuned through molecular electronic structure. To this end, we designed and synthesized **A-azo** and **AD-azo**, alongside **AA-azo** (Fig. 3A and figs. S4 and S5). Each crosslinker incorporates two carboxyl groups for nanocarrier construction. However, directly positioning both carboxyl groups at the para-sites strongly perturbs the azobenzene π-system and limits systematic modulation of electronic effects. To decouple crosslinking functionality from electronic regulation, we introduced a methylene spacer strategy in **A-azo** and **AD-azo**, in which one carboxyl group was connected to the azobenzene core through a ─CH_2_─ linker. This molecular series therefore establishes a controllable electronic gradient across the azobenzene core, enabling systematic modulation of azobenzene reduction kinetics.

**A-azo**, an electron-withdrawing structure, was synthesized by the Mill’s reaction of ethyl 4-nitrophenylacetate and 4-aminobenzoic acid, followed by hydrolysis (fig. S4). **AD-azo**, containing both electron-donating and electron-withdrawing substituents, was synthesized via diazotization of *N*-methyl-*N*-phenylglycine ethyl ester and subsequent coupling with 4-aminobenzoic acid, followed by hydrolysis (fig. S5). In this series, the carboxyl group functions as an electron-withdrawing unit (**A**), whereas the dimethylamino group acts as an electron donor (**D**). Both structures were confirmed by ^1^H- and ^13^C-NMR (figs. S6 to S9). This molecular series therefore establishes a controllable electronic gradient across the azobenzene core, enabling systematic regulation of electron-transfer competition within the reaction network.

Under severe hypoxia [O_2_ (<0.1 mg/liter)], we compared the enzymatic cleavage kinetics of three compounds in the azoR/NADPH system. Detection was performed at 340 nm for **AA-azo** and **A-azo** and at 462 nm for **AD-azo** to minimize interference from azoR or NADPH (fig. S10A). The *t*_1/2_ was 10.7 hours for **AA-azo**, 5.1 hours for **A-azo**, and 1.1 hours for **AD-azo**, spanning nearly one order of magnitude in reaction kinetics (fig. S11 and table S1). These results demonstrate that substituent electronic structure directly regulates productive electron transfer toward the azo bond, resulting in distinct reduction kinetics.

Substituent engineering also altered the sensitivity of the reaction sensitivity toward O_2_-mediated inhibition (Fig. 3B). Under mild hypoxia [O_2_ (∼5.3 mg/liter)], **AD-azo** exhibited relatively fast cleavage (*t*_1/2_ ≈ 3 hours), whereas **AA-azo** exhibited significantly slower reduction (*t*_1/2_ ≈ 32.5 hours). Under normoxia, **AD-azo** still showed 10 to 15% cleavage, whereas **A-azo** and **AA-azo** were nearly completely suppressed. Thus, substituent engineering alters not only intrinsic reduction kinetics but also the O_2_ sensitivity of the competing reaction network.

DFT calculations further revealed the mechanistic origin of these kinetic differences. The activation energy barriers (Δ*G*^†^) for electron transfer from FMNH^−^ to **AD-azo**, **A-azo**, and **AA-azo** were 9.8, 14.1, and 24.0 kcal/mol, respectively (Fig. 3D). This trend indicates that increasing electron-withdrawing character progressively decreases the electron affinity of the azo bond and raises the barrier for productive reduction. In all cases, the second electron transfer required more energy than the first, indicating it as the rate-limiting step (Fig. 3E). In parallel, intermediate stability analysis showed that the azo^−^ intermediate of **AA-azo** has the lowest activation barrier for oxidation by O_2_, making it most susceptible to O_2_-mediated reverse electron transfer (Fig. 3C). Together, these results show that substituent electronic structure simultaneously regulates forward electron transfer, intermediate stability, and O_2_-mediated back oxidation, thereby controlling electron partitioning between competing pathways. By tuning the azobenzene core from electron-withdrawing configurations (**AA-azo** and **A-azo**) to donor-acceptor (**AD-azo**) structures, both the magnitude and O_2_ sensitivity of the reduction kinetics can be continuously regulated. Consequently, continuous O_2_ gradients are translated into distinct kinetic outputs rather than simple binary activation states.

### Bioreduction kinetics program nanoscale disassembly and release pathways

After establishing that O_2_-partitioned electron transfer governs azobenzene reduction kinetics, we next investigated whether this competition-controlled molecular behavior could be translated into programmable nanoscale structural evolution and drug release. Using ampicillin (Amp), a broad-spectrum hydrophobic antibiotic, as a model drug, we electrostatically self-assembled three azobenzene crosslinkers with glycol chitosan (GC). (*25*) Amp was then encapsulated through ultrasound-assisted loading to generate **azo-GC**_**Amp**_ nanoparticles (NPs) (Fig. 4A). According to the incorporated crosslinkers, the resulting NPs were denoted **AA-GC**_**Amp**_, **A-GC**_**Amp**_, and **AD-GC**_**Amp**_. Through optimization of the [NH_2_]_GC_:[COOH]_azo_ ratio, a 2:1 ratio formed stable NPs with a hydrodynamic diameter of ∼200 nm and a surface potential of +10 mV (Fig. 4, B and C; fig. S12; and table S2). Transmission electron microscopy (TEM) confirmed a uniform spherical morphology with a dehydrated size of ∼150 nm (Fig. 4D). The NPs were stabilized by the balance between electrostatic interactions and hydrophobic assembly: The hydrophilic GC backbone maintained colloidal stability, whereas azobenzene moieties formed hydrophobic microdomains through π-π stacking and hydrophobic interactions. Ultraviolet-visible (UV-Vis) spectra showed characteristic absorption peaks at 340, 430, and 462 nm, confirming the retention of the chromophore during self-assembly (fig. S10B). The NPs maintained structural stability for over 30 days at 4°C and could be readily redispersed after lyophilization (fig. S13). In contrast, NPs prepared with a 1:1 or 1:2 ratio (300 to 400 nm, with negative or near-neutral surface charge) aggregated significantly within 48 hours due to disrupted charge balance. The azobenzene-rich hydrophobic domains not only enabled efficient loading of Amp (4 to 5%, table S2) but also created confined reaction-active microenvironments that enabled collective nanoparticle disassembly behavior (Fig. 4E).

**Fig. 4.**
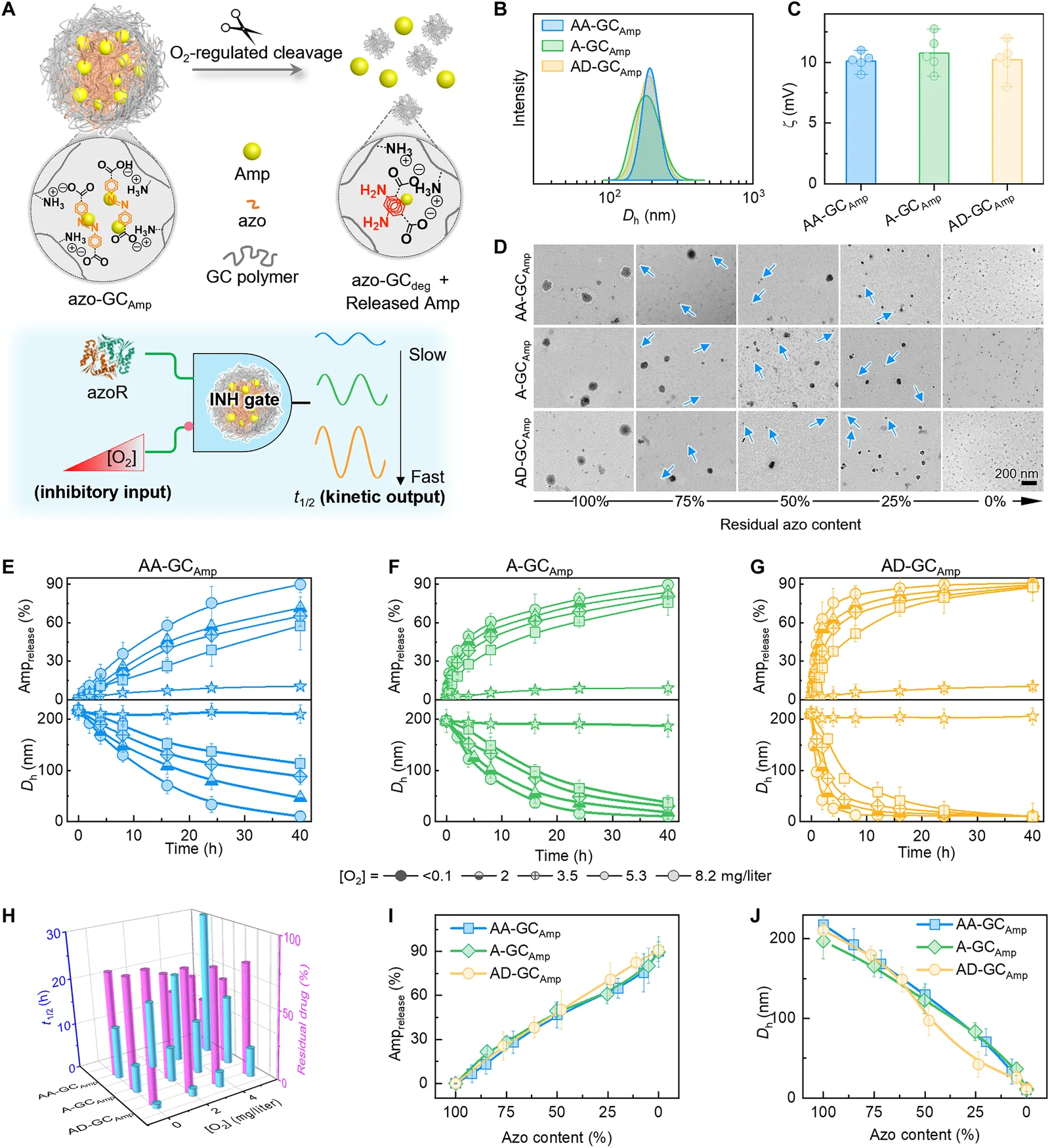
Bioreduction kinetics program nanoscale disassembly and release pathways. (**A**) Schematic illustration of the preparation of **azo-GC**_**Amp**_ NPs from GC and azo-based crosslinkers, followed by O_2_-regulated cleavage, nanoparticle dissociation, and Amp release. The resulting release behavior can be functionally interpreted as an INH-type kinetic response, in which O_2_ acts as an inhibitory input and cleavage kinetics determine the output magnitude. (**B**) Hydrodynamic size distributions and (**C**) zeta potentials of **azo-GC**_**Amp**_ NPs prepared at an [NH_2_]_GC_:[COOH]_azo_ ratio of 2:1. (**D**) TEM images of **azo-GC**_**Amp**_ NPs at different degradation stages corresponding to residual azobenzene contents of 100, 75, 50, 25, and 0%. Blue arrowheads indicate ultrafine **azo-GC**_**deg**_ generated during nanoparticle dissociation. (**E** to **G**) Time-dependent Amp release and hydrodynamic diameter changes of **azo-GC**_**Amp**_ NPs incubated with azoR/NADPH under different O_2_ concentrations (<0.1, 2, 3.5, 5.3, and 8.2 mg/liter). (**H**) Half-lives (*t*_1/2_) of Amp release and corresponding Amp release efficiencies under different O_2_ concentrations. (**I** and **J**) Correlations between residual azobenzene content and Amp release (I) or nanoparticle diameter (J). Data are presented as means ± SD (*n* = 3).

After confirming the structural stability of the nanocarriers, we examined whether molecular cleavage kinetics could be directly translated into macroscopic release behavior. As shown in Fig. 4 (E to G), **azo-GC**_Amp_ NPs displayed strongly O_2_-dependent release behavior in the azoR/NADPH system. Under severe hypoxia [O_2_ (<0.1 mg/liter)], all three NPs released ∼90% of Amp within 40 hours, indicating efficient activation of azobenzene cleavage. In contrast, under normoxia [O_2_ (8.2 mg/liter)], drug release remained below 5%, demonstrating strong O_2_-mediated inhibition. These results indicate that the O_2_-dependent molecular reduction behavior is preserved after translation into nanoscale assemblies.

The release kinetics further retained the substituent-dependent modulation behavior. Under severe hypoxia, the release *t*_1/2_ of **AA-GC**_**Amp**_, **A-GC**_**Amp**_, and **AD-GC**_**Amp**_ were 11.0, 5.3, and 1.4 hours, respectively, reflecting nearly an order of magnitude difference (Fig. 4H and fig. S14). **AA-GC**_**Amp**_, with a double electron-withdrawing structure and highest reduction barrier, showed the slowest release behavior, whereas **AD-GC**_**Amp**_, having the donor-acceptor configuration and lowest reduction barrier, showed the fastest response. This close correspondence demonstrates that molecular reduction kinetics remain conserved during translation to nanoscale release behavior.

The O_2_-dependent release profiles further revealed continuous kinetic modulation. As O_2_ levels increased from severe hypoxia [O_2_ (<0.1 mg/liter)] to mild hypoxia [O_2_ (2 to 5.3 mg/liter)], all NPs showed progressively slower release kinetics, although the degree of suppression differed substantially among structures. At O_2_ concentration of 5.3 mg/liter, **AD-GC**_**Amp**_’s *t*_1/2_ increased to ∼3 hours, while **AA-GC**_**Amp**_ slowed markedly to 32.7 hours, demonstrating a 23-fold kinetic window (Fig. 4H and table S3). The release profile closely paralleled the azobenzene degradation kinetics, indicating that antibiotic release is governed by competition-controlled molecular reduction kinetics rather than by passive carrier diffusion (Fig. 4, I and J). Notably, all three nanocarriers remained responsive under mild hypoxia [O_2_ (5.3 mg/liter)], suggesting comparable activation thresholds for hypoxia-triggered release. In contrast, their release rates differed markedly, indicating that programmable dosage control is governed primarily by modulation of kinetic output after activation rather than by differences in activation threshold.

Within this framework, O_2_ functions as an inhibitory input: High O_2_ levels intercept electron flux and suppress nanoparticle disassembly and drug release (output = OFF), whereas hypoxic conditions permit productive azo reduction and activate release behavior (output = ON). Meanwhile, substituent-controlled electron partitioning continuously tunes the amplitude and O_2_ sensitivity of the release response. In this way, the nanoscale release behavior can be functionally interpreted as an INH-type logic response with continuously modulatable outputs, while its fundamental origin remains rooted in competition-controlled molecular kinetics (Fig. 4A).

To further examine how molecular kinetics manifest as nanoscale structural evolution, we monitored nanoparticle size changes during enzymatic degradation. Dynamic light scattering (DLS) analysis showed progressive nanoparticle shrinkage during azobenzene cleavage (Fig. 4, E to G; fig. S14; and table S3). Under normoxic conditions [O_2_ (8.2 mg/liter)], nanoparticle size remained stable at ∼200 nm, consistent with an inhibited structural state. Under hypoxia, however, the carriers gradually dissociated into smaller assemblies. Specifically, under mild hypoxia [O_2_ (5.3 mg/liter)], **AD-GC**_**Amp**_ NPs shrank from 210 to ∼22 nm in 24 hours and fully dissociated into ∼10-nm particles within 40 hours. In contrast, **A-GC**_**Amp**_ and **AA-GC**_**Amp**_ degraded more slowly, reaching ∼37 and ∼113.5 nm after 40 hours, respectively. Under severe hypoxia [O_2_ (0.1 mg/liter)], all NPs ultimately degraded to ∼10-nm particles, with the size-transition kinetics closely matching both molecular cleavage kinetics and drug-release behavior.

This strong correspondence among cleavage *t*_1/2_, nanoparticle shrinkage, and release kinetics demonstrates a direct kinetic translation process spanning molecular electron transfer, nanoscale structural evolution, and macroscopic release behavior. Rather than acting as passive responsive carriers, the NPs amplify the underlying competition-controlled molecular kinetics into collective structural transitions across length scales. Consequently, the rate and timing of nanoparticle size transition can be continuously regulated under different O_2_ conditions. The emergence of the ∼10-nm assemblies suggests that azo cleavage does not lead to complete molecular dissolution. Instead, structural evolution proceeds through a reorganization pathway in which residual intermolecular interactions remain sufficient to maintain nanoscale assemblies.

The structural analysis of the fully degraded particles further provided insight into the origin of these ultrafine assemblies. Although azo cleavage disrupted the original crosslinked network, the degradation products retained aromatic structures that could potentially associate through hydrophobic and π-π interactions. Meanwhile, electrostatic interactions between the carboxyl groups of the degradation products and the amino groups of GC remained available. These residual intermolecular interactions likely promoted structural reorganization and reassembly into ultrafine NPs rather than complete molecular dissolution (fig. S15). ^1^H-NMR confirmed the coexistence of aniline groups and GC polymer chains within the degraded assemblies (fig. S16). Together with the DLS and TEM results, these observations suggest that the degradation products remain at least partially associated with GC chains within the ultrafine NPs.

Despite differing degradation kinetics, the three NPs exhibited similar size evolution and release patterns at equivalent azobenzene conversion levels. TEM analysis confirmed that NPs maintained spherical morphologies throughout degradation stages corresponding to 75, 50, 25, and 0% residual azobenzene content (Fig. 4D). This behavior suggests that nanoparticle evolution proceeds through progressive dissociation of the azobenzene-crosslinked network rather than catastrophic structural disruption. Substituent electronic effects therefore regulate the rate of network dissociation by controlling the underlying competition-governed electron-transfer kinetics. Overall, these results establish a direct kinetic linkage between molecular reduction behavior and programmable nanoparticle disassembly and drug-release kinetics.

### Oxygen-regulated kinetic drive infection-adaptive therapeutic responses

To evaluate how electron competition–controlled kinetics dynamically regulates therapeutic behavior under fluctuating hypoxia, we investigated how reaction kinetics translates into nanoscale structural evolution, drug release, and antibacterial activity. As shown in Fig. 5A, electron competition between O_2_ reduction and azo reduction generates reversible ON/OFF switching of nanoparticle activation. Hypoxia relieves O_2_-mediated electron consumption and permits azo cleavage, whereas O_2_-rich conditions suppress electron transfer toward the azo bond and inhibit nanoparticle disassembly. Consistent with this mechanism, **AD-GC**_**Amp**_ NPs exhibited reversible switching during four cycles between severe hypoxia [O_2_ (0.1 mg/liter)] and mild hypoxia [O_2_ (5.3 mg/liter)] (Fig. 5, B and C). Within 0.5 hours of transitioning to severe hypoxia, particle size decreased from ∼210 to 129 nm, with a ∼27% burst release of Amp. Upon returning to mild hypoxia, nanoparticle dissociation slowed markedly and cumulative release gradually increased to 57% over the following 6 hours. These results demonstrate that O_2_ functions not simply as a trigger but as a continuous regulator of electron flux partitioning between competing reduction pathways, thereby dynamically controlling nanoparticle disassembly and release kinetics.

**Fig. 5.**
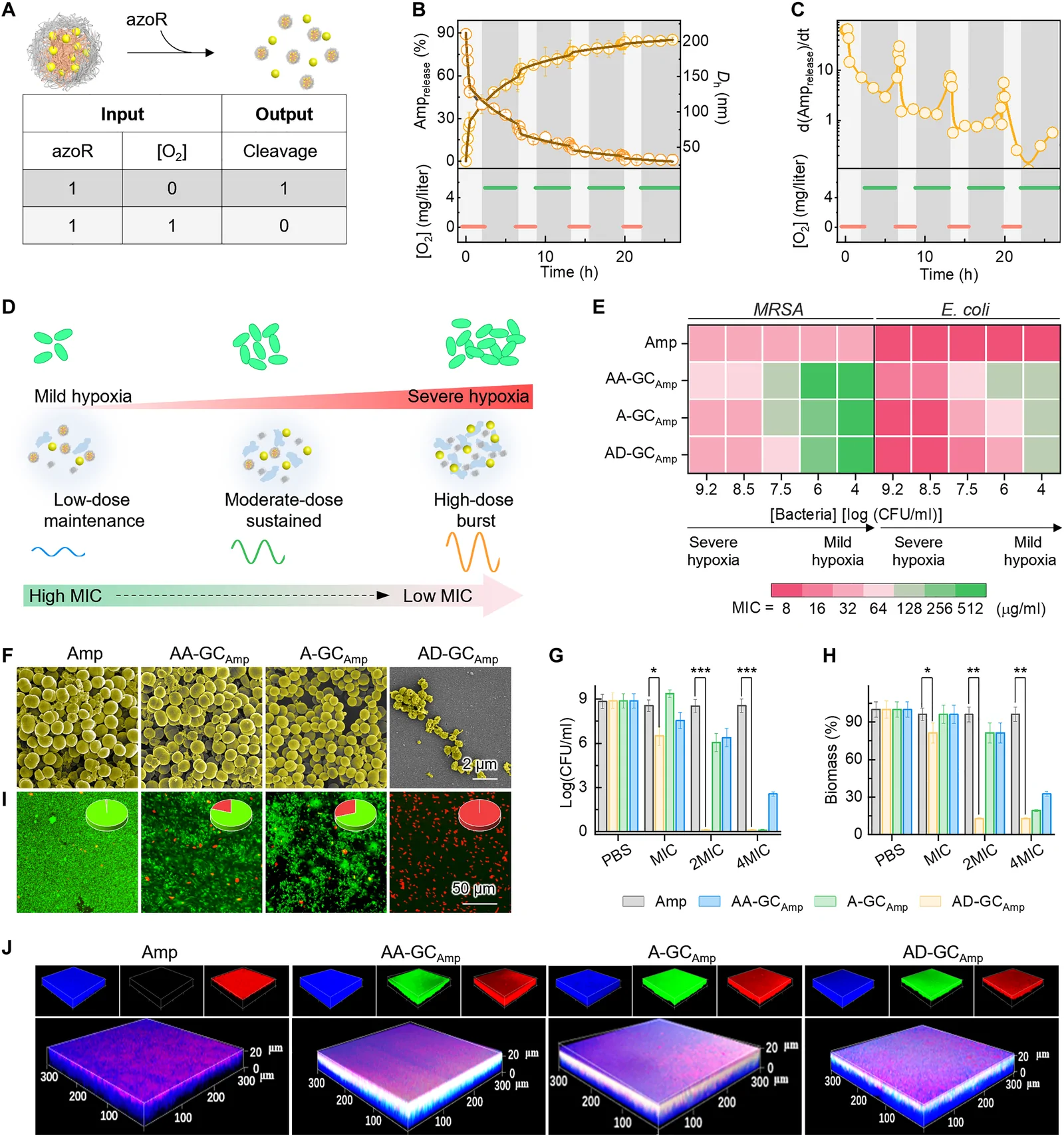
Kinetically programmed release behaviors enable adaptive antibacterial activity and biofilm eradication. (**A**) Schematic illustration of hypoxia-dependent structural evolution and release behaviors of **azo-GC**_**Amp**_ NPs. The accompanying truth table summarizes the dependence of azobenzene cleavage on azoR (activating input) and O_2_ (inhibitory input). (**B**) Time-dependent Amp release and hydrodynamic diameter changes of **AD-GC**_**Amp**_ NPs during four cycles of alternating O_2_ concentrations (<0.1 and 5.3 mg/liter). (**C**) Differential release profiles derived from the Amp-release curves shown in (B). (**D**) Schematic summary of kinetically programmed therapeutic outputs, showing low-dose maintenance release under mild hypoxia, moderate-dose sustained release under intermediate hypoxia, and high-dose burst release under severe hypoxia. (**E**) Minimum inhibitory concentrations (MICs) of **azo-GC**_**Amp**_ NPs and free Amp against MRSA and *E. coli* at different bacterial inoculum densities. (**F**) SEM images of MRSA biofilms after 24-hour treatment with **azo-GC**_**Amp**_ NPs at 4MIC (based on the MIC of Amp). Free Amp was used as control. (**G** and **H**) Viable bacteria counts (G) and biomass quantification (H) of MRSA biofilms after 24-hour treatment with **azo-GC**_**Amp**_ NPs at different concentrations. PBS and free Amp were used as controls. Data are presented as means ± SD (*n* = 3). (**I**) CLSM images of live/dead-stained MRSA biofilms after 24-hour treatment with **azo-GC**_**Amp**_ NPs at 4MIC. Live and dead bacteria are shown in green and red, respectively. (**J**) CLSM images of MRSA biofilms after 30-min incubation with rhodamine-labeled Amp (red) and fluorescein-labeled **azo-GC**_**Amp**_ NPs (green) at 2MIC. Bacterial nuclei were stained with Hoechst 33342 (blue).

This dynamic behavior was programmable through substituent electronics. Although **A-GC**_**Amp**_ and **AA-GC**_**Amp**_ also showed similar switching behavior, their response speed and magnitude differed due to distinct electron-transfer kinetics. During severe hypoxia, all NPs released significantly more drug than under mild hypoxia, following the order **AD** > **A** > **AA**. After four cycles, **AD-GC**_**Amp**_ NPs nearly depleted their drug load, whereas **AA-GC**_**Amp**_ NPs still retained ∼40% of the encapsulated drug (fig. S17). These results demonstrate that substituent electronics continuously regulate therapeutic output under fluctuating O_2_ conditions.

After establishing the dynamic switching mechanism, we next examined whether bacterial metabolism could directly reshape therapeutic behavior through modulation of the reaction network. Real-time O_2_ electrode data showed that increasing bacterial concentration [Gram-positive Methicillin-resistant *Staphylococcus aureus* (MRSA) and Gram-negative *Escherichia coli* (*E. coli*)] from 1 × 10^4^ to 1.6 × 10^9^ colony-forming units (CFU)/ml progressively reduced O_2_ levels from normoxia (∼8.2 mg/liter) to severe hypoxia (<0.1 mg/liter; fig. S18). Thus, bacterial burden continuously reshapes local O_2_ availability and thereby modulates electron partitioning between O_2_ reduction and azo reduction pathways.

Antibacterial assays further demonstrated infection-adaptive therapeutic behavior emerging from this kinetic framework. Under severe hypoxia (1.6 × 10^9^ CFU/ml), **AD-GC**_**Amp**_ and **A-GC**_**Amp**_ NPs showed minimum inhibitory concentration (MIC) values comparable to free Amp, consistent with rapid burst release and high drug output following relief of O_2_-mediated inhibition (Fig. 5, D and E, and table S4). In contrast, **AA-GC**_**Amp**_ NPs displayed higher MIC values because its slower reduction kinetics generated lower release output under the same conditions. Under mild hypoxia (1 × 10^4^ CFU/ml), all NPs showed significantly higher MIC values (128 to 512 μg/ml), consistent with maintenance-release behavior and reduced drug output. Notably, substituent electronics continuously tuned release intensity under identical bacterial concentrations, resulting in distinct MIC profiles across different infection states. These results indicate that MIC variation directly reflects the analog release output programmed by electron-competition kinetics. Consequently, therapeutic intensity can be dynamically matched to infection severity through O_2_-regulated control of release behavior.

Bacterial biofilms provide a more stringent infection microenvironment in which high cell density and restricted O_2_ diffusion generate persistent hypoxia. These conditions continuously relieve O_2_-mediated inhibition of azo reduction, enabling sustained nanoparticle activation, disassembly, and drug release throughout the biofilm matrix. Therefore, biofilms offer an ideal setting to evaluate how hypoxia-responsive release behavior translates into spatially controlled therapeutic delivery. Antibacterial testing revealed significant differences in biofilm eradication efficacy among the three nanocarrier systems. At 2MIC (Amp’s MIC), **AD-GC**_**Amp**_ NPs completely cleared up to eight orders of magnitude of bacterial load in MRSA and *E. coli* biofilms, while crystal violet staining and scanning electron microscopy (SEM) showed ∼90% biofilm disruption (Fig. 5, F to H, and figs. S19 to S23). In contrast, **AA-GC**_**Amp**_ NPs at 4MIC reduced bacterial load by only ∼3 orders of magnitude and left ∼30% of the biofilm intact. Live/dead fluorescence microscopy further confirmed extensive bacterial killing in **AD-GC**_**Amp**_–treated biofilms, whereas slow-response nanoparticle groups retained significant green fluorescence signals corresponding to surviving bacteria (Fig. 5I and fig. S24). Confocal laser scanning microscopy (CLSM) revealed that **AD-GC**_**Amp**_ NPs penetrated through the entire biofilm depth, allowing efficient codelivery of the carrier and antibiotic, whereas **A-GC**_**Amp**_ and **AA-GC**_**Amp**_ NPs showed limited penetration (Fig. 5J).

The distinct antibiofilm activities can be rationalized by the interplay between hypoxia-responsive activation and substituent-controlled release kinetics. Although persistent biofilm hypoxia permits activation of all carrier systems, the subsequent structural evolution remains highly dependent on the kinetic landscape encoded by substituent electronics. **AD-GC**_**Amp**_ NPs underwent rapid disassembly, drug release, and size reduction upon exposure to the biofilm surface, quickly transitioning from ∼200-nm particles into ultrafine nanoscale assemblies. This accelerated size evolution facilitated deeper diffusion through the dense extracellular matrix and enhanced antibiotic delivery into hypoxic biofilm interiors. In contrast, **AA-GC**_**Amp**_ NPs exhibited slower activation, delayed size transition, and reduced penetration efficiency, leading to weaker biofilm clearance. Thus, biofilms not only provide a hypoxic environment that favors nanoparticle activation but also amplify the kinetic differences encoded by substituent electronics. Collectively, these findings demonstrate that hypoxia-responsive release behavior can be translated across scales into adaptive therapeutic performance, linking molecular reduction kinetics with nanoparticle evolution, biofilm penetration, and antibiofilm efficacy.

### Electronic regulation of metabolite reactivity programs toxicological outcomes

After establishing the electronically programmable reaction framework, we next investigated how substituent-controlled electronic structure governs the reactivity and safety of the degradation products. A critical challenge lies in mitigating the toxicity of aniline derivatives generated upon reduction. The International Agency for Research on Cancer (IARC) classifies azobenzene (group 3) as noncarcinogenic, but its enzymatic reduction products—aniline and some derivatives (group 2A)—pose significant toxicity risks (*19*). This toxicity arises from metabolic activation, producing reactive aryl diazonium ions and reactive oxygen species 0(ROS), leading to oxidative stress, mitochondrial damage, hemolysis, methemoglobinemia, genotoxicity, and tissue damage through protein binding (Fig. 6A). These downstream biological consequences are fundamentally governed by metabolite electronic structure and redox reactivity. Thus, within our O_2_-partitioned electron-transfer framework, electronic structure not only determines electron competition and cleavage kinetics but also dictates the reactivity of the resulting metabolites. This establishes a unified reaction-consequence engineering framework in which the same electronic principle simultaneously regulates reaction kinetics, metabolite activation, and biosafety. We therefore implemented a dual-level strategy: At the molecular level, substituent electronics were used to regulate metabolite redox reactivity, while at the nanostructural level, carrier confinement further restricted biological activation (Fig. 6A).

**Fig. 6.**
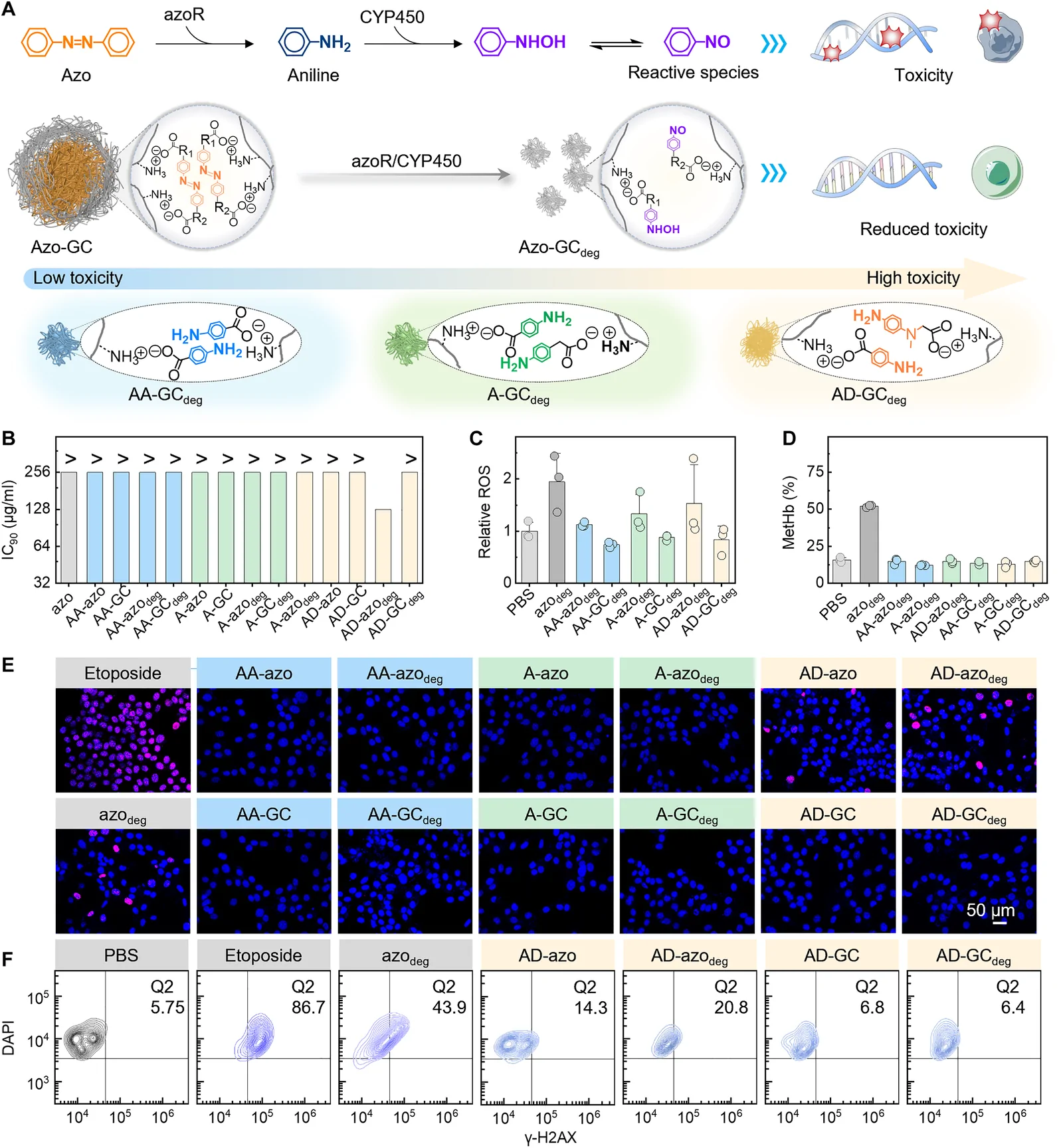
Nanoconfinement and substituent engineering jointly regulate the toxicity of azobenzene degradation products. (**A**) Schematic illustration of toxicity regulation by nanoparticle confinement and substituent engineering. Nanoconfinement reduces the toxicity of azobenzene degradation products, while substituent electronic structures enable further tuning of toxicity levels. (**B**) IC_90_ values of azo-based crosslinkers, **azo-GC** NPs, and their degradation products against 3T3 fibroblast cells. (**C**) MetHb formation and (**D**) intracellular reactive oxygen species generation in 3T3 cells induced by liver microsome-treated degradation products of azo-based crosslinkers and ultrasmall particles derived from **azo-GC** NPs. Data are presented as means ± SD (*n* = 3). (**E**) Representative CLSM images of 3T3 cells treated with azo-based crosslinkers and ultrasmall particles, stained with 4′,6-diamidino-2-phenylindole (DAPI) (blue), and γ-H2AX (red). (**F**) Flow cytometry analysis of γ-H2AX expression in 3T3 cells after treatment with **AD-azo**, **AD-azo**_**deg**_, **AD-GC**, and **AD-GC**_**deg**_ NPs. The concentration of aniline-derived species in the degradation products was fixed at 256 μg/ml. Etoposide and **azo**_**deg**_ were used as positive controls, and PBS served as the negative control.

To validate this mechanism, we fully degraded the azobenzene derivatives under severe hypoxia [O_2_ (<0.1 mg/liter)] using the azoR/NADPH system, obtaining degradation products (**AD-azo**_**deg**_, **A-azo**_**deg**_, and **AA-azo**_**deg**_) and corresponding nanocarrier degradation particles (**AD-GC**_**deg**_, **A-GC**_**deg**_, and **AA-GC**_**deg**_), with **azo**_**deg**_ as a control (fig. S25). Hemolysis (hemolytic concentration at 10% hemolysis >1024 μg/ml) and cytotoxicity assays (inhibitory concentration at 90%: 128 μg/ml for **AD-azo**_**deg**_ and >256 μg/ml for others) indicated good biocompatibility for all crosslinkers, NPs, and their degradation products (Fig. 6B and figs. S26 and S27). However, these bulk toxicity measurements cannot capture the underlying electronically regulated metabolic activation processes that ultimately determine oxidative and genotoxic risk.

We therefore co-incubated the degradation products with liver microsomes to simulate metabolic activation, measuring methemoglobin (MetHb) formation in whole blood and ROS generation in 3T3 cells. **Azo**_**deg**_ induced a ∼50% increase in MetHb and ∼200% increase in ROS, confirming the strong oxidative and hematotoxicity of traditional azobenzene metabolites (Fig. 6, C and D). The toxicity outputs followed a clear substituent-dependent electronic trend. Degradation products from all crosslinkers and NPs induced MetHb formation similar to the negative control. Only **AD-azo**_**deg**_ metabolites increased ROS by 30 to 50%, suggesting residual metabolic activation and oxidative stress. **AA-azo**_**deg**_, with electron-withdrawing substituents at both ends, showed no significant ROS increase. These results demonstrate that substituent-controlled electron density directly regulates metabolic activation pathways. Electron-withdrawing substituents reduce aromatic electron density, suppress electron transfer–mediated redox activation, and thereby inhibit ROS generation at the source. Notably, nanoparticle-derived degradation particles (**AD-GC**_**deg**_, **A-GC**_**deg**_, and **AA-GC**_**deg**_) showed slightly lower ROS levels than the control, suggesting that embedding azobenzene crosslinkers within the chitosan matrix limits enzyme accessibility, reducing oxidative risk. This additional nanostructural confinement effect reveals that reaction consequences can be further regulated beyond molecular electronic design alone.

After evaluating oxidative and hematotoxicity, we assessed the genotoxic potential of azobenzene crosslinkers, **azo-GC** NPs, and their degradation products using immunofluorescence and CLSM. After metabolic activation with liver microsomes, γ-H2AX staining was used to detect DNA double-strand breaks in 3T3 cells. As shown in Fig. 6E, etoposide, the positive control, induced strong red fluorescence, confirming severe DNA damage. **Azo**_**deg**_ also produced notable γ-H2AX signals, although weaker than etoposide, indicating significant genotoxic risk. In contrast, the azobenzene crosslinkers and their degradation products exhibited improved genotoxic safety. **AD-azo** and **AD-azo**_**deg**_ resulted in significantly lower γ-H2AX signals, while the electron-withdrawing **AA** and **A** derivatives showed fluorescence similar to the negative control, indicating minimal DNA damage.

All **azo-GC** NPs and their degradation particles showed negligible γ-H2AX signals, consistent with the previous ROS and MetHb data. Flow cytometry analysis provided further evidence (Fig. 6F). Etoposide induced 86.7% γ-H2AX–positive cells, while **azo**_**deg**_ showed 43.9%, highlighting the genotoxic potential of traditional azobenzene. **AD-azo** and **AD-azo**_**deg**_ reduced the positive cell rate to 14.3 and 20.8%, respectively, showing the beneficial effect of substituent modulation. Notably, the nanocarriers, **AD-GC** NPs and their degradation products (**AD-azo**_**deg**_), reduced DNA damage to near background levels (∼6.5%) comparable to the negative control. Together, these results establish a direct structure-reaction consequence relationship in which substituent-controlled electron transfer governs not only cleavage kinetics but also metabolite-induced oxidative and genotoxic outputs.

To further evaluate in vivo biosafety, **azo-GC** NPs and their degradation products were intravenously injected into mice. After 24 hours, blood was collected via retro-orbital puncture, and liver and kidney function, leukocyte subtypes, red blood cell (RBC) indices, and platelet parameters were assessed. As shown in fig. S28, all blood biochemical and cellular indices remained within normal physiological ranges, with no significant differences from the phosphate-buffered saline (PBS) control group, indicating no acute hemolysis, liver or kidney damage, or systemic inflammatory responses. After 7 days, the histological analysis of major organs via hematoxylin and eosin (H&E) staining further confirmed the absence of cell damage, inflammatory infiltration, or necrosis in the liver, kidneys, or other vital organs (fig. S29). These in vivo results demonstrate that electronically regulated reaction pathways can be translated into favorable systemic biosafety profiles.

Collectively, these results reveal that the electronic structure of azobenzene simultaneously governs therapeutic kinetics and toxicological activation. Electron-withdrawing substituents not only reshape electron partitioning during azo reduction but also suppress the redox activation of the resulting metabolites, thereby reducing the formation of toxic intermediates. In parallel, nanoscale confinement provides an additional level of regulation by limiting the accessibility of degradation products to metabolic enzymes. Consequently, therapeutic behavior and biosafety are controlled through the same electron-transfer framework across molecular and nanostructural levels, rather than through independent optimization processes. This coupling establishes electronically programmed reaction networks as a strategy for simultaneously regulating functional output and biological safety in responsive therapeutic systems (Fig. 6A).

### Kinetic programming enables context-dependent therapeutic adaption

We next investigated whether O_2_-partitioned electron-transfer kinetics could directly program therapeutic behavior across distinct infection environments. To this end, we established two representative models with fundamentally different O_2_ landscapes: an acute pulmonary infection characterized by severe hypoxia and a chronic wound infection with mild hypoxia (Fig. 7, A and F) (*26*, *27*). Within this framework, O_2_ no longer acts merely as a trigger for nanoparticle activation, but as a kinetic regulator that continuously reshapes therapeutic outputs across different pathological states. In the acute MRSA pulmonary infection model, mice were injected with **AD-GC**_**Amp**_, **A-GC**_**Amp**_, or **AA-GC**_**Amp**_ NPs 3 hours postinfection, with PBS and free Amp groups as controls (Fig. 7A). All nanoparticle-treated groups showed markedly improved antibacterial efficacy compared with free Amp (Fig. 7B). Among them, **AD-GC**_**Amp**_ NPs achieved the best efficacy, reducing bacterial load to ∼10^5^ CFU per lung, corresponding to an approximately five-order reduction relative to PBS and a two-order reduction relative to free Amp. In contrast, **A-GC**_**Amp**_ and **AA-GC**_**Amp**_NPs showed progressively reduced antibacterial effects (∼10^6^ and 3 × 10^6^ CFU per lung, respectively). Similar trends were observed in bronchoalveolar lavage fluid (fig. S30).

**Fig. 7.**
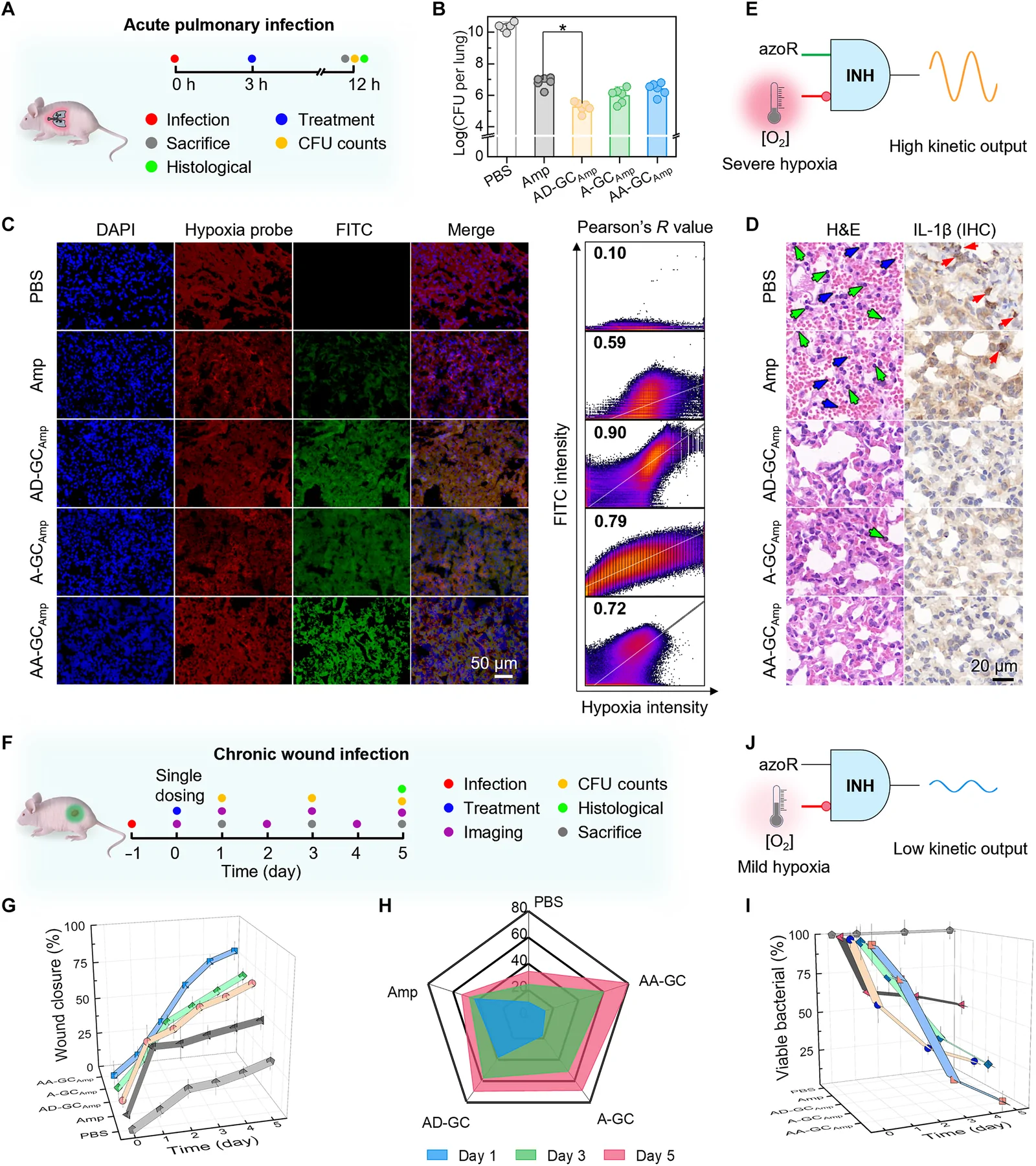
Kinetically programmed release behaviors enable infection-adaptive therapeutic responses. (**A**) Timeline illustrating the progression of an acute MRSA pulmonary infection model and the subsequent treatment schedule. (**B**) Quantification of viable bacteria in lung tissues after treatment with different therapies. (**C**) Distribution of hypoxia signals (red) and FITC signals (green) in lung tissues, together with the corresponding Pearson’s correlation coefficients. Pulmonary cells were stained with Hoechst 33342 (blue). (**D**) Representative H&E and IL-1β–stained lung sections collected at the experimental end point. Irregular cells are indicated by blue arrowheads, and inflammatory cell infiltration is indicated by green arrowheads. The dosage of Amp in each treatment was fixed at 15 mg/kg body weight. (**E**) Schematic illustration of an INH-type kinetic response under severe hypoxia, resulting in a high kinetic output. (**F**) Timeline illustrating the progression of a chronic *E. coli* wound infection model and the subsequent treatment schedule. (**G**) Relative wound area over time, (**H**) wound-closure profiles, and (**I**) viable bacterial percentages in wound tissues following different treatments. PBS- and Amp-treated groups were used as controls. The dosage of Amp in each treatment was fixed at 20 μl of a 4 mg/ml solution per mouse. (**J**) Schematic illustration of an INH-type kinetic response under mild hypoxia, resulting in a low kinetic output.

Immunofluorescence staining revealed extensive hypoxic regions in infected lung tissues, as indicated by strong red fluorescence signals (Fig. 7C). Correspondingly, azobenzene-based NPs preferentially accumulated in hypoxic areas, with intensity following the order: **AD-GC**_**Amp**_ > **A-GC**_**Amp**_ > **AA-GC**_**Amp**_. Histological analysis revealed that **AD-GC**_**Amp**_ NPs most effectively reduced interleukin-1β (IL-1β) levels and preserved alveolar integrity, indicating superior inflammation control and tissue protection.

These results demonstrate that severe hypoxia selectively favors systems with rapid electron-transfer kinetics. **AD-GC**_**Amp**_ NPs, having the lowest reduction barrier, rapidly underwent cleavage-driven structural dissociation and nanoparticle shrinkage within the severely hypoxic infection core (Fig. 7E). This accelerated structural evolution enabled earlier generation of smaller NPs with enhanced tissue and biofilm penetration capability, ultimately improving antibacterial efficacy in regions that are typically difficult to access by diffusion-limited therapies.

We then evaluated the system in a mild hypoxia *E. coli* wound infection model, where therapeutic efficacy depends more strongly on sustained release behavior than rapid activation (Fig. 7F). Under these conditions, **AD-GC**_**Amp**_ NPs produced rapid initial antibacterial effects, achieving ∼38% wound closure on day 1, comparable to free Amp (Fig. 7, G to I, and fig. S31). However, their effect diminished over time. In contrast, **AA-GC**_**Amp**_ NPs showed slower initial therapeutic responses (13 to 18% closure on day 1) but progressively improved wound healing and achieved the best therapeutic outcome by day 5.

These observations indicate that under mild hypoxia, slower electron-transfer kinetics become advantageous because they sustain drug availability over extended treatment periods (Fig. 7J). Consequently, rapid-response **AD**-type systems exhibit a characteristic “burst depletion” profile, whereas slow-response **AA**-type systems maintain prolonged therapeutic activity because of their higher activation barriers and slower cleavage kinetics. Together, these results demonstrate that substituent-controlled electron partitioning governs not only release amplitude but also the temporal profile of therapeutic activity. By programming reduction kinetics at the molecular level, the system dynamically adapts therapeutic outputs to distinct pathological O_2_ landscapes, thereby linking electron-transfer chemistry with infection-adaptive treatment outcomes (Fig. 1).

Pharmacokinetic and biodistribution studies showed that all three nanocarriers exhibited highly similar circulation profiles and organ distribution patterns (figs. S32 and S33). In addition, histological examination revealed no detectable tissue abnormalities in major organs following treatment (Fig. 7D and figs. S34 and S35), indicating comparable in vivo biosafety across all formulations. These observations suggest that systemic exposure, biodistribution, and biosafety differences are unlikely to be the major factors governing therapeutic outcome. Instead, the distinct performances observed in acute and chronic infection models are more consistently explained by the programmed kinetic outputs encoded through substituent-controlled electron partitioning.

## DISCUSSION

Rather than functioning through conventional binary hypoxia triggering, the adaptive therapeutic behavior observed in this system originates from O_2_-partitioned electron-transfer kinetics within a competing reaction network. In the azobenzene bioreduction pathway, O_2_ acts not merely as an external environmental cue but as a competing electron acceptor that redistributes electron flux between productive azo reduction and O_2_ reduction pathways. This competition converts O_2_ gradients from simple activation signals into kinetic regulatory variables that directly govern reaction permissibility, reduction rate, nanoparticle disassembly, and therapeutic output.

Within this framework, substituent electronic structure serves as a programmable molecular parameter that reshapes electron-transfer barriers, intermediate stability, and O_2_ sensitivity, thereby encoding distinct kinetic behaviors across different pathological environments. Under severe hypoxia, reduced O_2_ competition permits rapid electron transfer, accelerated nanoparticle disassembly, and burst antibacterial activity. Under mild hypoxia, persistent O_2_ interception suppresses electron flux toward azo reduction, resulting in slower structural evolution and sustained therapeutic release. Consequently, therapeutic adaptation emerges not from discrete environmental switching but from continuous kinetic partitioning within the reaction network.

The kinetic regulation established here propagates hierarchically across multiple length scales, spanning molecular electron transfer, nanoscale structural evolution, drug-release dynamics, and infection-level therapeutic responses. In this process, molecular electron-transfer kinetics are amplified into collective nanoscale disassembly behavior, enabling chemical reaction dynamics to directly dictate material evolution and therapeutic functionality. In parallel, the same electronically regulated framework also governs downstream metabolite reactivity and toxicological outcomes, linking therapeutic function and biosafety through a unified reaction-consequence relationship. These findings therefore establish O_2_-partitioned electron competition as a chemically programmable mechanism for converting continuous physiological gradients into adaptive functional outputs.

More broadly, this work suggests that biologically relevant environmental variables can be incorporated directly into reaction networks as kinetic regulators rather than treated solely as external triggers. By coupling reaction-network competition with programmable molecular electronic structure, this framework provides a generalizable strategy for constructing chemically adaptive materials capable of dynamically translating physiological gradients into tunable structural and therapeutic behaviors.

## MATERIALS AND METHODS

### Ethical statement

This study complies with all applicable ethical regulations. All animal experiments were approved by the Institutional Animal Care and Use Committee of Tongji Medical College, Huazhong University of Science and Technology (approval no. TJH-202412032). Animal procedures were conducted in accordance with the institutional guidelines for the care and use of laboratory animals.

### Materials

Ethyl bromoacetate, *N*-methylaniline, 4-aminobenzoic acid, 4-aminophenylacetic acid, 4-nitrophenylacetic acid, 4-nitrobenzoic acid, glucose anhydrous, sodium nitrite, sodium carbonate, sodium sulfate, sodium chloride (NaCl), sodium dithionite (Na_2_S_2_O_4_), magnesium sulfate, ammonium chloride (NH_4_Cl), zinc powder, iron (III) chloride hexahydrate (FeCl_3_·6H_2_O), lithium hydroxide monohydrate (LiOH·H_2_O), sodium hydroxide, triethylamine, hydrochloric acid (HCl, 37%), sulfuric acid (98%), acetic acid, 2-methoxyethanol, methanol (MeOH), ethanol (EtOH), ethyl acetate (EtOAc), dichloromethane (DCM), *n*-hexane, GC (weight-average molecular weight of 100 kDa, 87.5% deacetylated), glutaric dialdehyde, crystal violet, ampicillin (*Amp*), etoposide, microsome rat liver pool male, β-nicotinamide adenine dinucleotide 2′-phosphate reduced tetrasodium salt hydrate (NADPH), 4′,6-diamidino-2-phenylindole, enzyme azoreductase, and methemoglobin assay kit from Shanghai Aladdin Reagent Company were used as purchased without further purification. Dulbecco’s modified Eagle’s medium, penicillin-streptomycin liquid, trypsin, fetal bovine serum, and PBS were purchased from Thermo Fisher Scientific (Waltham, MA, USA). Dichlorodihydrofluorescein diacetate (DCFH-DA), cell counting kit-8 (CCK-8), and molecular probes (Hoechst 33342, LysoTracker Red) were obtained from Beyotime Biotechnology (Shanghai, China). MRSA American Type Culture Collection (ATCC) 43300 (MRSA) and *E. coli* ATCC 25922 (*E. coli*) were provided by Shanghai Luwei Technology Co. Ltd. NIH-3T3 mouse embryonic fibroblast cell line was originally purchased from ATCC. All reagents were used as received without any further purification, unless otherwise stated.

### Synthetic protocols of azo-based crosslinkers

#### *Synthesis of AA*-*azo*

**AA-azo** was synthesized according to a reported procedure using glucose-mediated reductive coupling of 4-nitrobenzoic acid under alkaline aqueous conditions at 70°C for 12 hours (*28*). After purification by recrystallization, **AA-azo** was obtained in 75.3% yield. ^1^H-NMR [δ, parts per million (ppm), 400 MHz, DMSO-*d*_6_]: 8.17(d, 4H, Ar-***H***), 8.01 (d, 4H, Ar-***H***).

#### *Synthesis of A*-*azo*

**A-azo** was synthesized from 4-nitrophenylacetic acid through esterification, nitrosobenzene-mediated Mills coupling with 4-aminobenzoic acid, and subsequent ester hydrolysis according to reported procedures (*28*–*31*). The final product was obtained in 54.9% yield after alkaline hydrolysis and acidification. ^1^H-NMR (δ, ppm, 400 MHz, DMSO-*d*_6_): 8.08 (2H, Ar-***H***), 7.90 (2H, Ar-***H***), 7.83 (2H, Ar-***H***), 7.45 (2H, Ar-***H***), 3.66 (2H, C***H***_**2**_COOH). ^13^C-NMR (δ, ppm, 100 MHz, DMSO-*d*_6_): 172.53, 167.31, 154.80, 151.17, 140.30, 133.02, 132.01, 131.98, 123.25, 122.95, 40.89.

#### *Synthesis of AD*-*azo*

**AD-azo** was synthesized through azo coupling of diazotized 4-aminobenzoic acid with ethyl *N*-methyl-*N*-phenylglycinate (compound **3**), followed by ester hydrolysis according to reported procedures (*31*–*33*). The electron-donating *N*-methylamino substituent was introduced to generate the donor-acceptor (AD) azobenzene structure. The final product was obtained in 55.4% yield. ^1^H-NMR (δ, ppm, 400 MHz, DMSO-*d*_6_): 8.08 (2H, Ar-***H***), 7.84 (4H, Ar-***H***), 6.84 (2H, Ar-***H***), 4.28 (2H, C***H***_**2**_COOH), 3.12 (3H, NC***H***_**3**_). ^13^C-NMR (δ, ppm, 100 MHz, DMSO-*d*_6_): 172.09, 167.10, 155.34, 152.83, 143.34, 131.91, 131.36, 125.49, 122.44, 112.38, 53.65, 29.76.

### Preparation of azo_deg_

**AA-azo**_**deg**_, **A-azo**_**deg**_, and **AD-azo**_**deg**_ were generated by azoreductase-mediated cleavage under severe hypoxia ([O_2_] < 0.1%). Briefly, azo-based crosslinkers (256 μg/ml) were incubated with azoreductase (1 mg/ml) and NADPH (1 mM) in PBS (pH 7.4) at 37°C until complete disappearance of the characteristic azobenzene absorption bands (300 to 500 nm). The resulting degradation products were isolated by lyophilization. For NMR characterization, sodium dithionite was used as a chemical reductant to generate the corresponding cleavage products following an analogous reduction procedure.

### Oxygen-regulated bioreduction kinetics of azo-based crosslinkers

Azoreductase-mediated cleavage of azo-based crosslinkers was performed in PBS (pH 7.4) containing azo crosslinkers (256 μg/ml), azoreductase (1 mg/ml), and NADPH (1 mM). Prior to reaction, the azoreductase solution was equilibrated under defined O_2_ concentrations using N_2_/O_2_ gas mixtures at 37°C. Reactions were conducted in a hypoxia chamber as illustrated in fig. S2, and aliquots were withdrawn at predetermined time points and exposed to air to terminate further reduction. Azo cleavage was monitored by UV-Vis spectroscopy. Degradation kinetics were fitted using OriginPro 2024 according to a second-order kinetic model (1/*y* = 1/*y*_0_ + *kt*, where *k* is the rate constant and *t* is the reaction time), and the half-life was calculated as *t*_1/2_ = 1/(*ky*_0_).

### Computational details

All DFT calculations were carried out using the Gaussian 16 Revision C.01 software package. Geometry optimizations and vibrational frequency analyses of all structures were performed at the B3LYP/6-31+G(d) level of theory. Solvent effects were considered using the SMD implicit solvation model with water as the solvent, and Grimme’s DFT-D3 dispersion correction with Becke-Johnson (BJ) damping was included throughout the calculations.

The activation free energy (Δ*G*^†^) for the electron-transfer process was estimated according to Marcus theory using the equation Δ*G*^†^ = (Δ*G* + λ)^2^/4λ, where Δ*G*^†^, Δ*G*, and λ represent the activation energy barrier, Gibbs free energy change, and reorganization energy, respectively (*34*–*38*). To determine these parameters, the geometries of the azo-based molecules and FMN-based species were first optimized, followed by frequency calculations to obtain their Gibbs free energies for evaluation of Δ*G*. Single-point energy calculations were subsequently performed to estimate the reorganization energy (λ), which was then used to calculate the corresponding electron-transfer energy barriers.

### Preparation of azo-based NPs and degradation assemblies

**Azo-GC**_**Amp**_ NPs were prepared by nanoprecipitation-assisted self-assembly of GC, azo-based dicarboxylic crosslinkers, and ampicillin (Amp). GC and azo crosslinkers were assembled at [NH_2_]_GC_ to [COOH]_azo_ molar ratios of 2:1, 1:1, or 1:2, followed by purification through centrifugal ultrafiltration (MWCO 3500 Da).

Ultrasmall degradation particles (**azo-GC**_**deg**_) were obtained by complete azoreductase/NADPH-mediated cleavage of azo-crosslinked GC NPs (**azo-GC** NPs without Amp loading) under severe hypoxia ([O_2_] < 0.1%). IR820-loaded and Cy5.5-loaded NPs were prepared using the same protocol, with Amp replaced by the corresponding cargo during nanoparticle assembly.

### MIC assay

MIC values were determined by the broth microdilution method. Bacterial suspensions (2 × 10^6^ CFU/ml) were incubated with serially diluted **azo-GC**_**Amp**_ NPs or Amp in TSB at 37°C for 24 hours. Bacterial growth was quantified by measuring the optical density at 600, and the inhibition percentage was calculated relative to uninoculated and untreated controls. The MIC was defined as the lowest concentration that inhibited >90% of bacterial growth.

### Hemolysis assay

Hemolytic activity of **azo**-based NPs and Amp was assessed using sheep RBCs. After incubation with test samples for 2 hours in PBS (pH 7.4), hemoglobin release was quantified by absorbance at 540 nm. Hemolysis was calculated relative to PBS-treated and water-lysed RBC controls. HC_10_ was defined as the minimum concentration causing >10% hemolysis.

### Cell viability assay

Cell viability was assessed using NIH-3T3 fibroblasts and a CCK-8 assay. Cells were exposed to Amp, GC, **azo**-based crosslinkers, or NPs for 24 hours under normoxic conditions ([O_2_] = 8.2 mg/liter). Metabolic activity was quantified by absorbance at 450 nm and normalized to untreated controls to determine relative cell viability.

### Methemoglobin formation assay

MetHb formation was assessed after microsomal activation of azobenzene-derived metabolites. **Azo**_**deg**_ or **azo-GC**_**deg**_ samples (equivalent to an aniline-derived species of 256 μg/ml) were incubated with liver microsomes (0.5 mg/ml) and NADPH (1 mM) in PBS (pH 7.4) at 37°C, followed by exposure to sheep whole blood. MetHb levels were quantified using a commercial assay kit. **Azo**_**deg**_ derived from unsubstituted azobenzene served as the positive control, and PBS served as the negative control.

### ROS generation assay

ROS generation induced by microsomally activated azobenzene metabolites was assessed in NIH-3 T3 cells using DCFH-DA staining. Cells were incubated for 24 hours with metabolized **azo**_**deg**_ or **azo-GC**_**deg**_ samples (equivalent to an aniline-derived species of 256 μg/ml), and intracellular ROS levels were quantified from DCF fluorescence. **azo**_**deg**_ derived from unsubstituted azobenzene served as the positive control, whereas PBS served as the negative control.

### Genotoxicity evaluation

Genotoxicity induced by microsomally activated azobenzene metabolites was assessed by γ-H2AX staining. NIH-3T3 cells were treated for 24 hours with metabolized azobenzene crosslinkers, **azo-GC** NPs, or their corresponding degradation products (equivalent to an aniline-derived species of 256 μg/ml). Etoposide and **azo**_**deg**_ derived from unsubstituted azobenzene served as positive controls. γ-H2AX expression was analyzed by confocal microscopy and flow cytometry to quantify DNA damage.

### Biofilm eradication assay

Biofilm biomass was evaluated by crystal violet staining, whereas viable bacteria remaining in mature biofilms were quantified by CFU enumeration. Three-day biofilms were treated with serial dilutions of **azo-GC**_**Amp**_ NPs or ampicillin for 24 hours. Biofilm biomass was determined from absorbance at 595 nm after crystal violet staining, and bacterial viability was assessed by colony counting following biofilm disruption. Untreated biofilms were used as controls.

### Biofilm imaging

Biofilm accumulation and penetration were evaluated by CLSM using Hoechst-stained biofilms incubated with rhodamine-labeled ampicillin (Amp-Rho) or FITC/rhodamine dual-labeled NPs. Biofilm morphology after treatment was examined by field emission–SEM, and three-dimensional biofilm structures were visualized by CLSM.

### Pharmacokinetic study

Healthy male C57BL/6 mice were intravenously injected with Cy5.5-loaded azo-GC NPs (15 mg/kg). At predetermined time points, blood samples were collected from the orbital venous plexus and diluted with deionized water. Fluorescence intensity was measured (λ_ex_ = 675 nm and λ_em_ = 710 nm), and blood concentrations were calculated using a standard calibration curve. Pharmacokinetic parameters were obtained by fitting the concentration-time profiles using Origin software.

### In vivo fluorescence imaging and biodistribution

Healthy male C57BL/6 mice were intravenously injected with IR820-loaded azo-GC NPs (15 mg/kg). At predetermined time points, whole-body fluorescence imaging was performed using a NIR-II imaging system (LS-NIR II-VIVO, LUSTER LightTech Co. Ltd., China) under 808-nm excitation with a 900-nm long-pass emission filter. At 24 hours postinjection, major organs were harvested for ex vivo fluorescence imaging.

### In vivo infection models

Acute pulmonary infection was established by intrapulmonary inoculation of MRSA (2 × 10^8^ CFU per mouse), whereas chronic wound infection was established by topical inoculation of *E. coli* (2 × 10^5^ CFU per wound). Infected animals received a single treatment with PBS, Amp, or **azo-GC**_**Amp**_ NPs. Bacterial burden, tissue pathology, and therapeutic outcomes were evaluated at the indicated time points.

### In vivo toxicity evaluation

Female Sprague-Dawley rats were intravenously administered **azo** crosslinkers or their corresponding degradation products (15 mg/kg). At 24 hours posttreatment, hematological parameters, serum biochemical markers, and histological changes in major organs were evaluated to assess systemic toxicity.

### Statistical analysis

Data are presented as means ± SD. Statistical analyses were performed using OriginPro 2024. Comparisons between two groups were conducted using Student’s *t* test, whereas multiple-group comparisons were analyzed by one-way analysis of variance (ANOVA). All statistical data were collected from experiments with at least 3 three replicates (*n* ≥ 3). The alpha value is *P < 0.05, **P < 0.01, ***P < 0.001, and ****P < 0.0001. n.s. indicates no significant difference.
