# Supplementary material for: Electron competition in azobenzene programs infection-adaptive antibacterial therapy
Source: Sci Adv. 2026 Nov 6;12(45):eaee7761. doi: 10.1126/sciadv.aee7761 (PMC13742143; doi:10.1126/sciadv.aee7761)
Supplement: Supplementary file 1 [file sciadv.aee7761_sm.pdf]

Science Advances
AAAS

Supplementary Materials for

Electron competition in azobenzene programs infection-adaptive antibacterial therapy

Yongjin Hu *et al.*

Corresponding author: Jingyi Rao, jrao@hust.edu.cn

Sci. Adv. **12**, eaee7761 (2026)
DOI: 10.1126/sciadv.aee7761

The PDF file includes:

Figs. S1 to S35
Tables S1 to S4
Legend for data S1

Other Supplementary Material for this manuscript includes the following:

Data S1

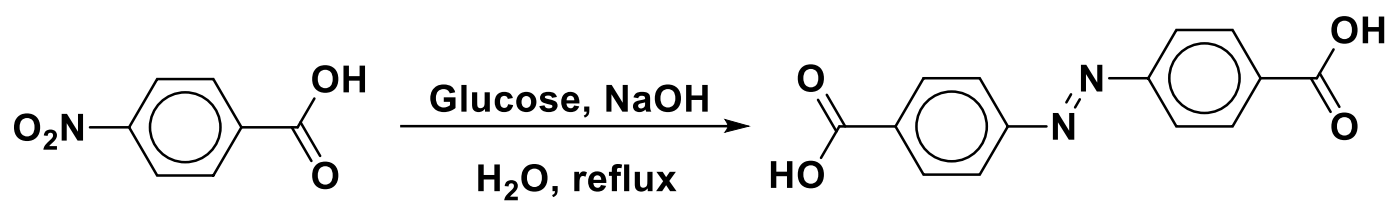

Figure S1. Synthetic pathway for the **AA-azo** crosslinker.

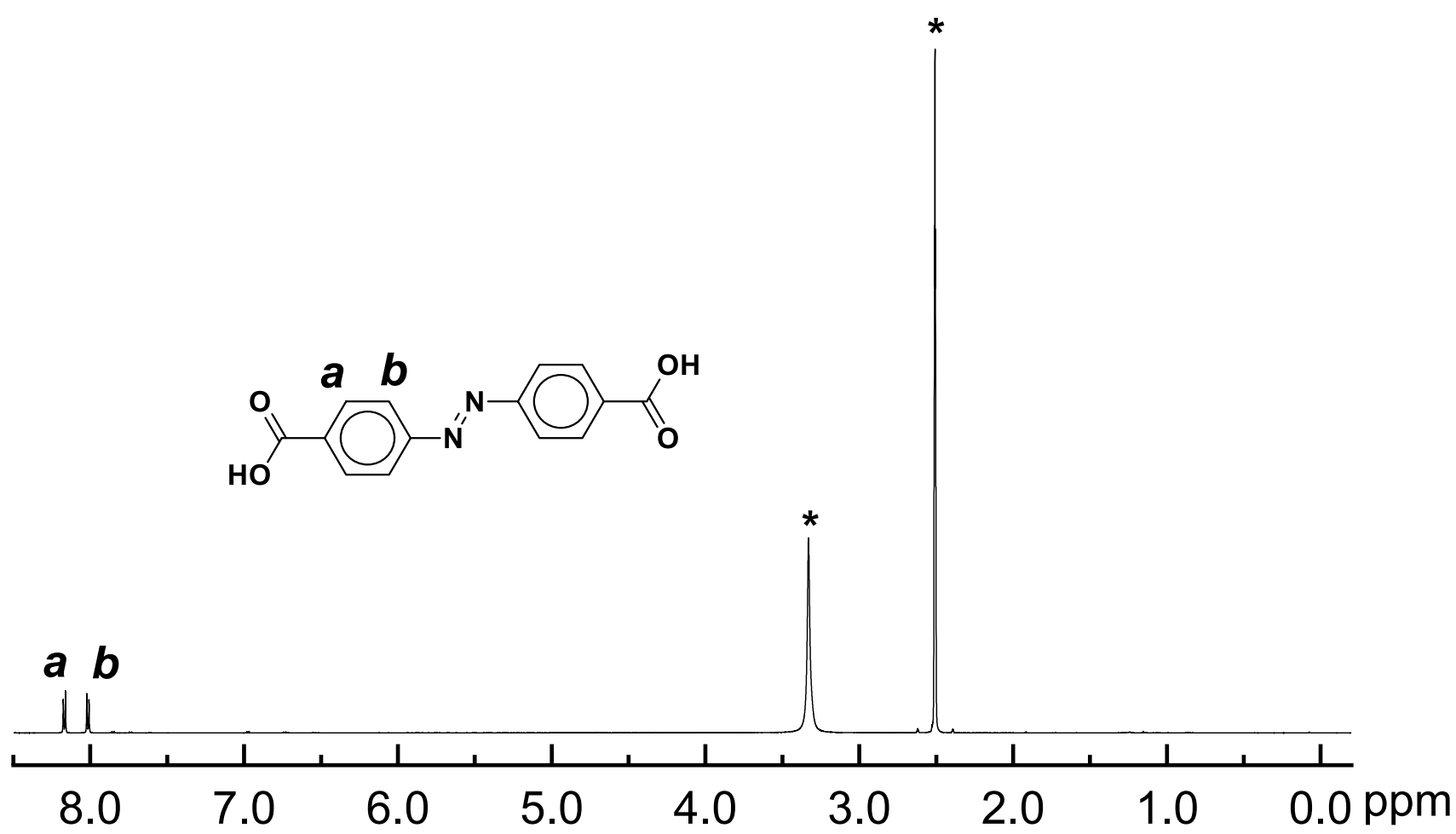

Figure S2. ^{1}H NMR spectrum of **AA-azo** in DMSO-d_6. Peaks corresponding to solvents are indicated with asterisks.

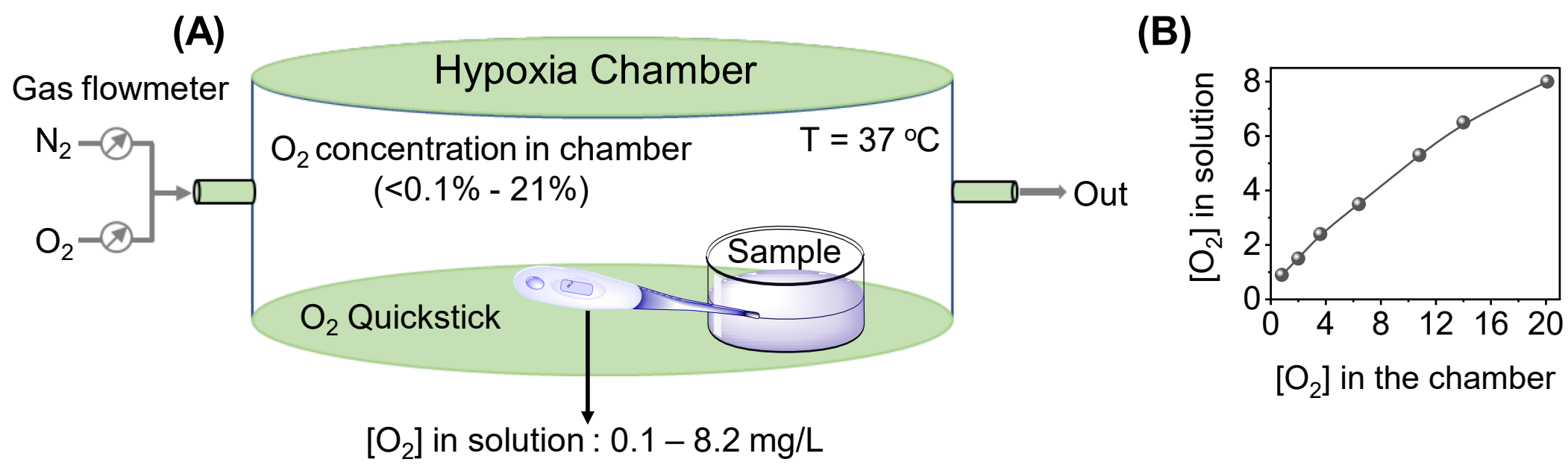

Figure S3. Schematic representation of the hypoxia incubator chamber. (A) The hypoxic degradation of azo-based crosslinkers and nanoparticles in solution was performed within the chamber, where oxygen levels were controlled to regulate the dissolved oxygen concentration in the solution. Under normoxic conditions (ambient oxygen concentration of 21%), the dissolved oxygen concentration, measured using an oxygen probe, was 8.2 mg/L. In contrast, under fully hypoxic conditions (ambient oxygen concentration <0.1%), the dissolved oxygen concentration was reduced to <0.1 mg/L; (B) Correlation between oxygen concentration in the hypoxia chamber and its equilibrium concentration in solution.

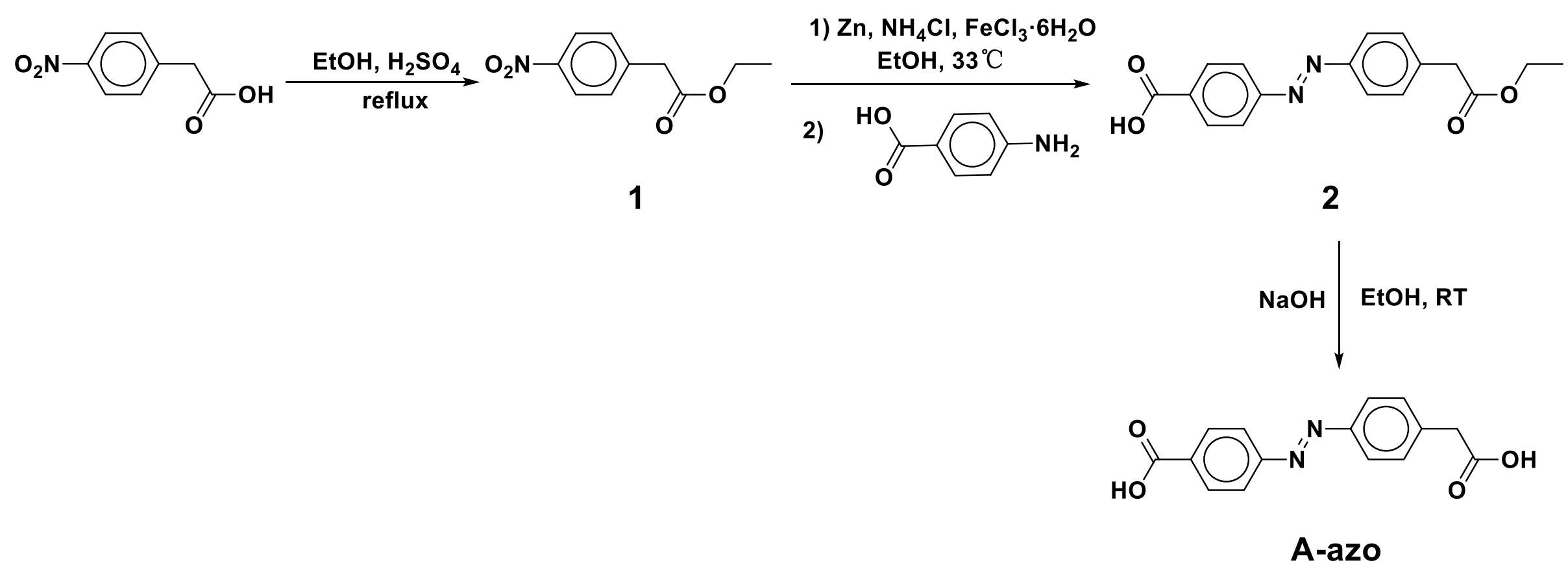

Figure S4. Synthetic pathway for the **A-azo** crosslinker.

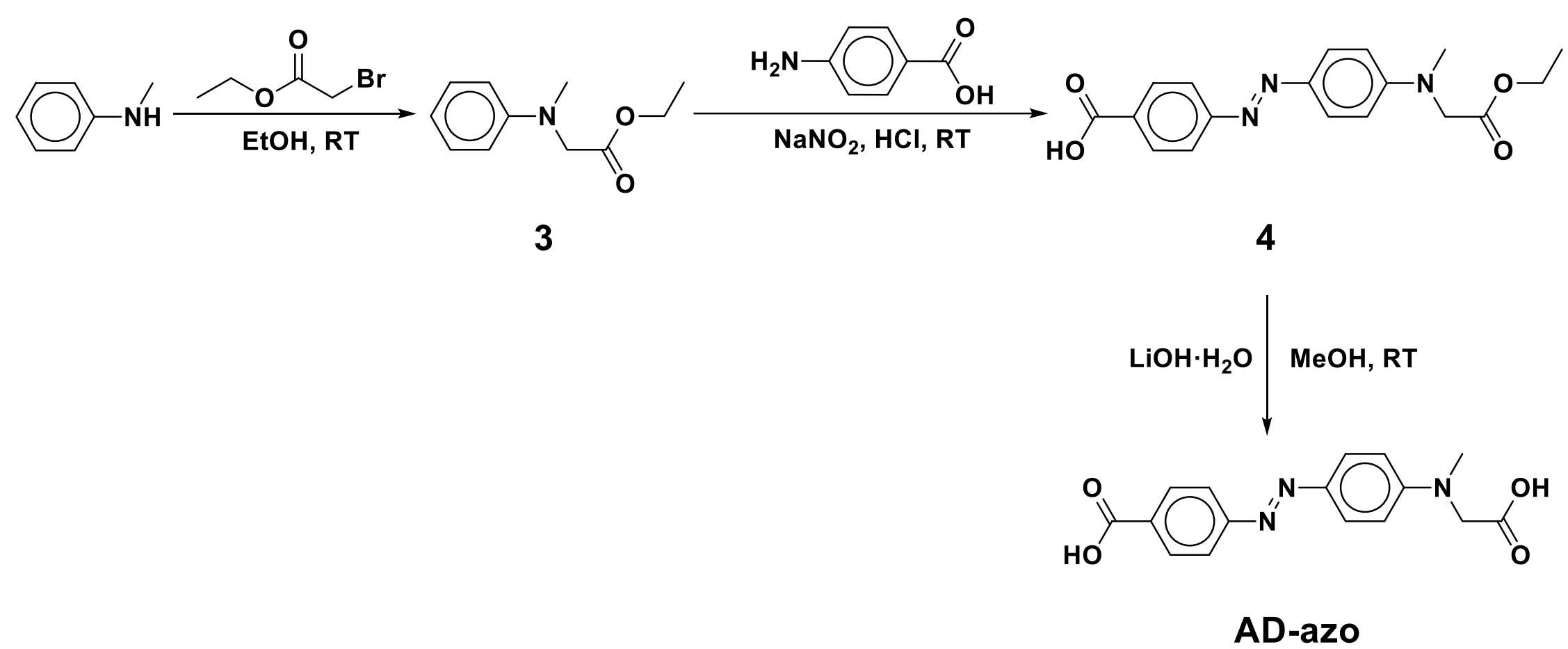

Figure S5. Synthetic pathway for the **AD-azo** crosslinker.

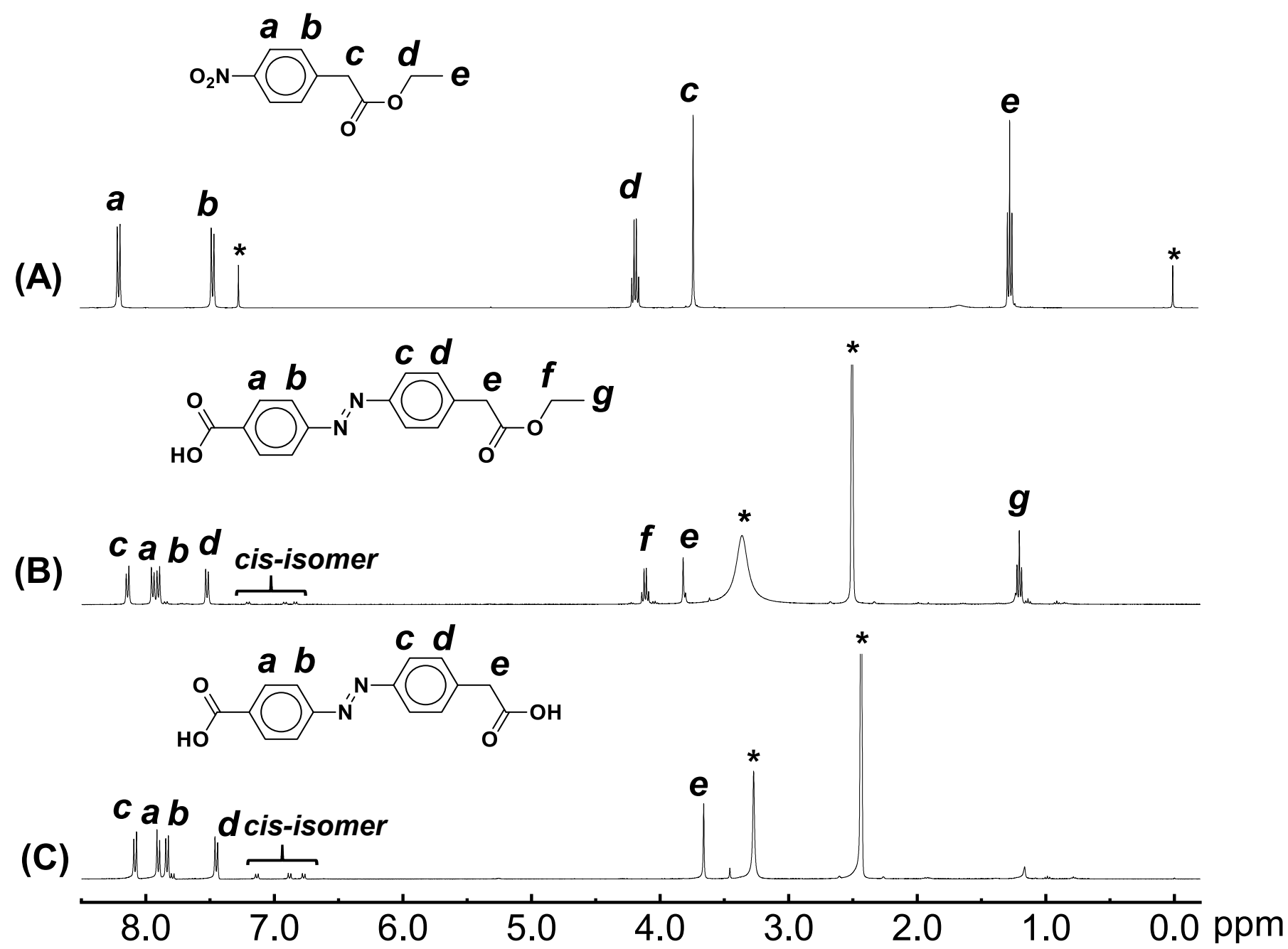

Figure S6. ^{1}H NMR spectra of (A) compound **1** in $CDCl_3$, (B) compound **2** in DMSO-d_6, and (C) **A-azo** in DMSO-d_6. Peaks corresponding to solvents and tetramethylsilane are indicated with asterisks.

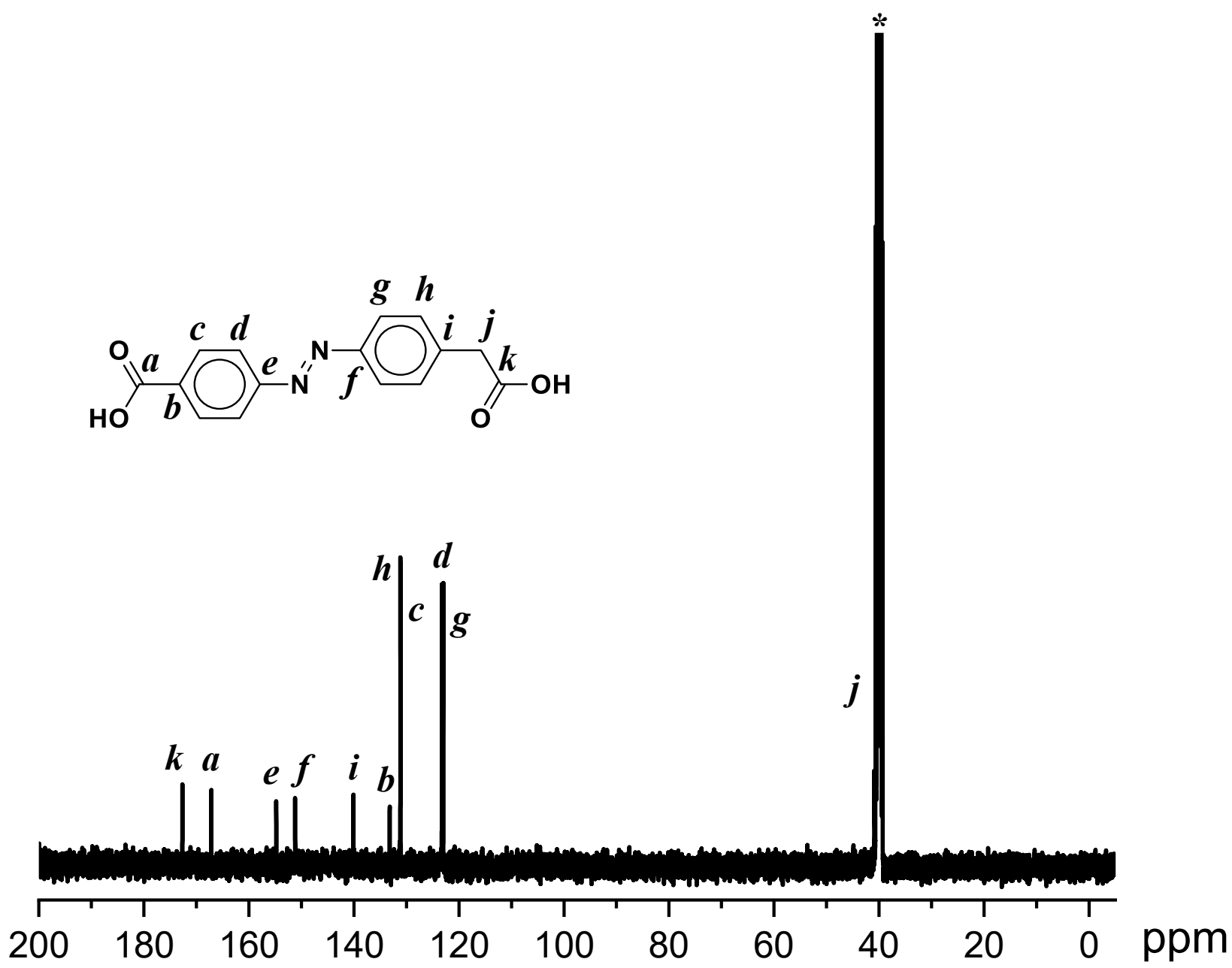

Figure S7. ^{13}C NMR spectrum of **A-azo** in DMSO-d_6. Peaks corresponding to solvents are indicated with asterisks.

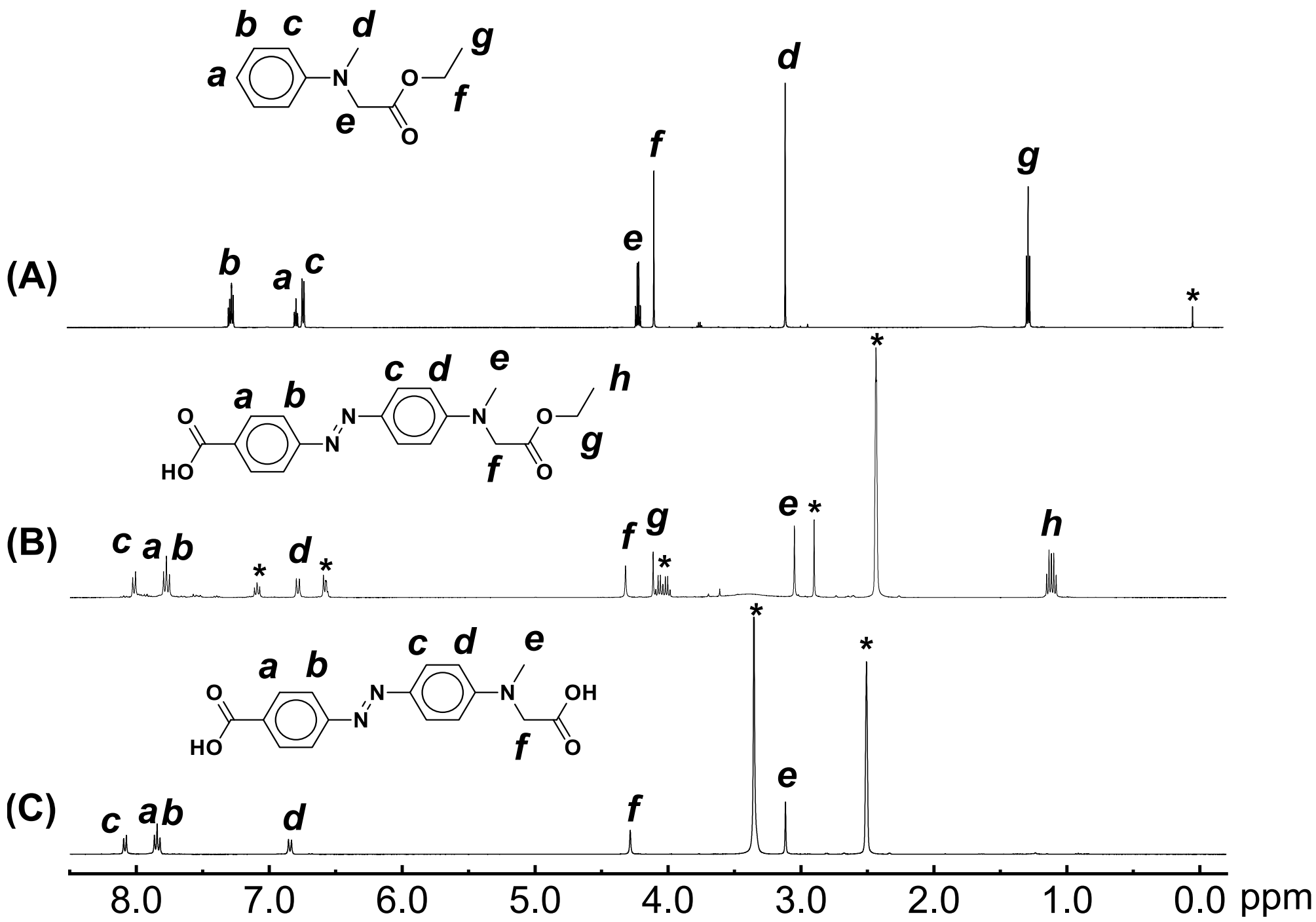

Figure S8. ^{1}H NMR spectra of (A) compound **3** in $CDCl_3$, (B) compound **4** in DMSO-d_6, and (C) **AD-azo** in DMSO-d_6. Peaks corresponding to solvents and tetramethylsilane are indicated with asterisks.

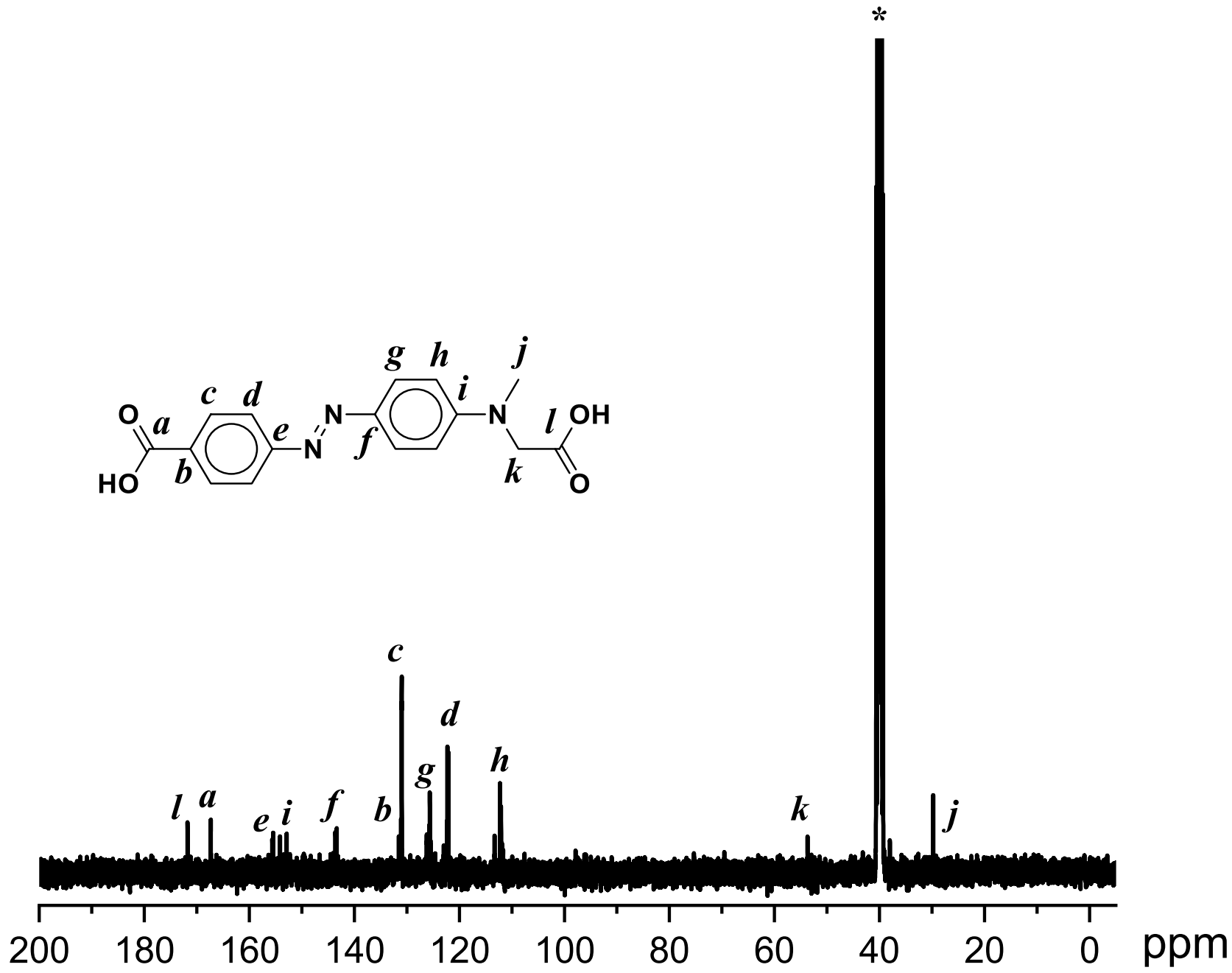

Figure S9. ^{13}C NMR spectra of **AD-azo** in DMSO-d_6. Peaks corresponding to solvents are indicated with asterisks.

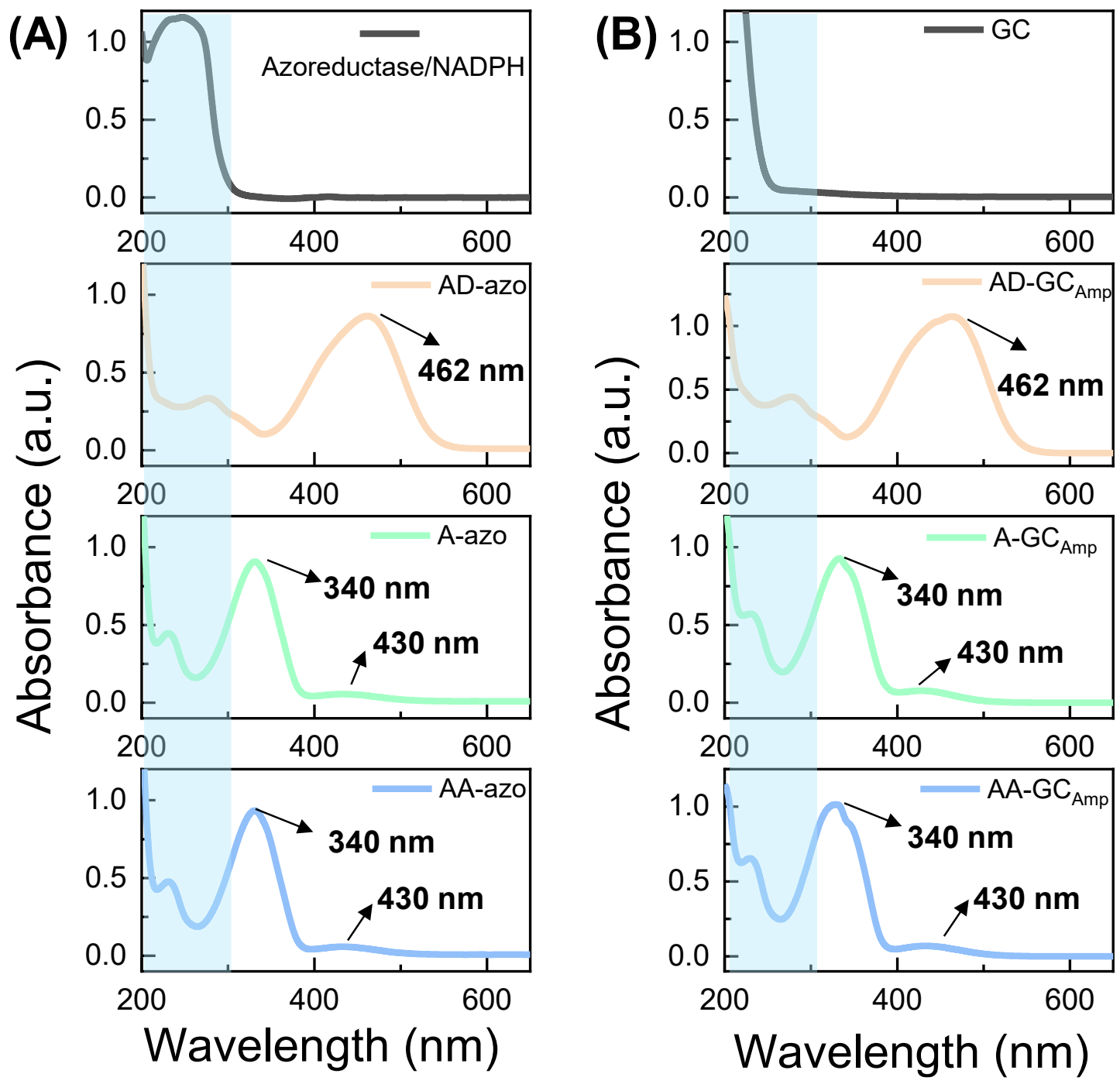

Figure S10. UV-Vis spectra of (A) azoreductase with NADPH and azo-based crosslinkers, and (B) GC and **azo-GC$_{\mathbf{Amp}}$** NPs in aqueous solutions.

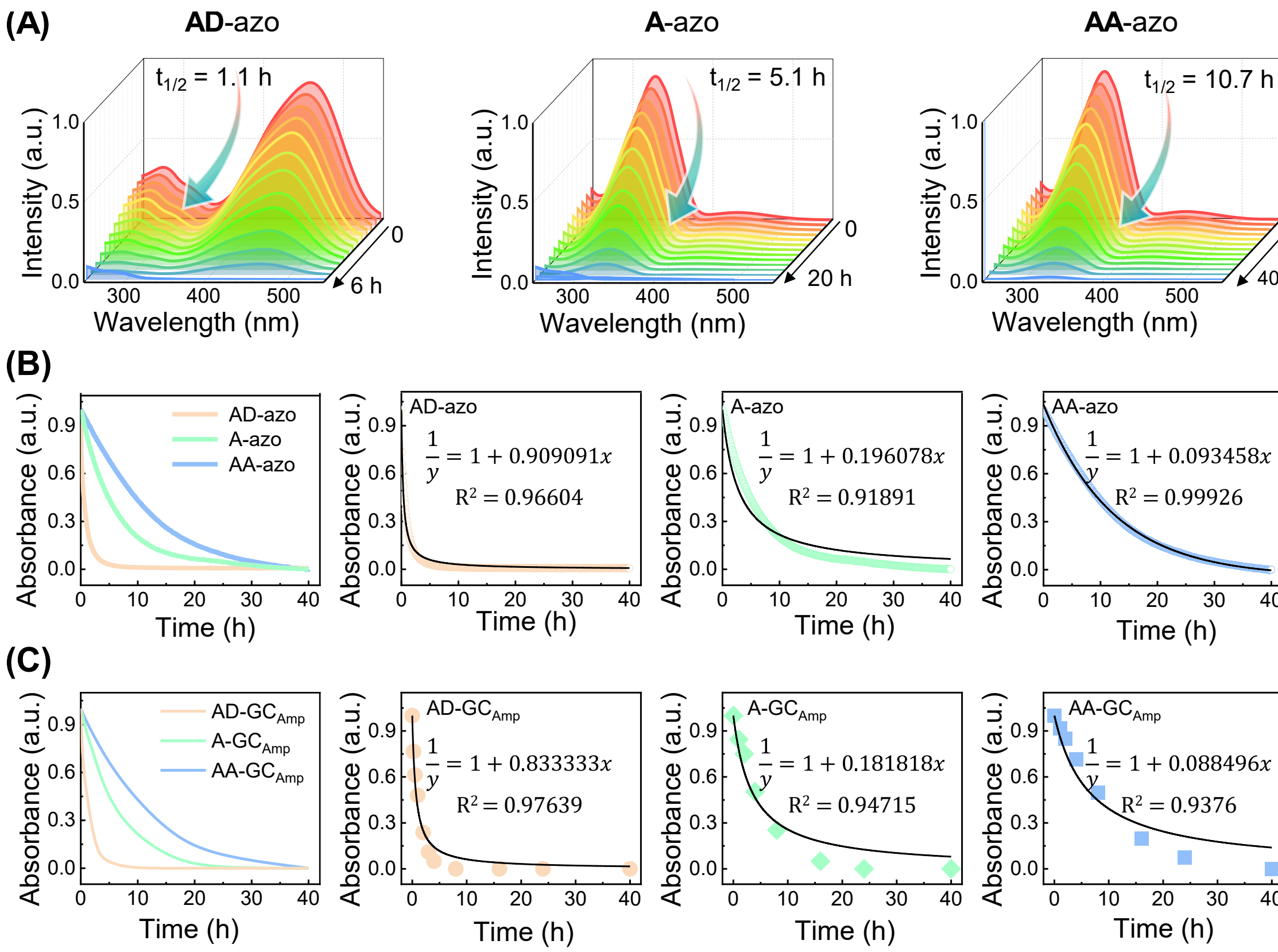

Figure S11. (A) Time-dependent UV-Vis spectral changes of azo-based crosslinkers in the presence of azoreductase/NADPH under hypoxic conditions ([O_2] <0.1 mg/mL). (B) Time-dependent UV-Vis absorption change and kinetics fittings of azo-based crosslinkers and (C) **azo-GC$_{\mathbf{Amp}}$** NPs in the presence of azoreductase/NADPH under the same hypoxic conditions. The kinetics were fitted using the equation $1/y = 1/y_0 + kt$ in OriginPro 2024, where k, t, and y_0 represent the rate constant, time, and initial y-axis value, respectively.

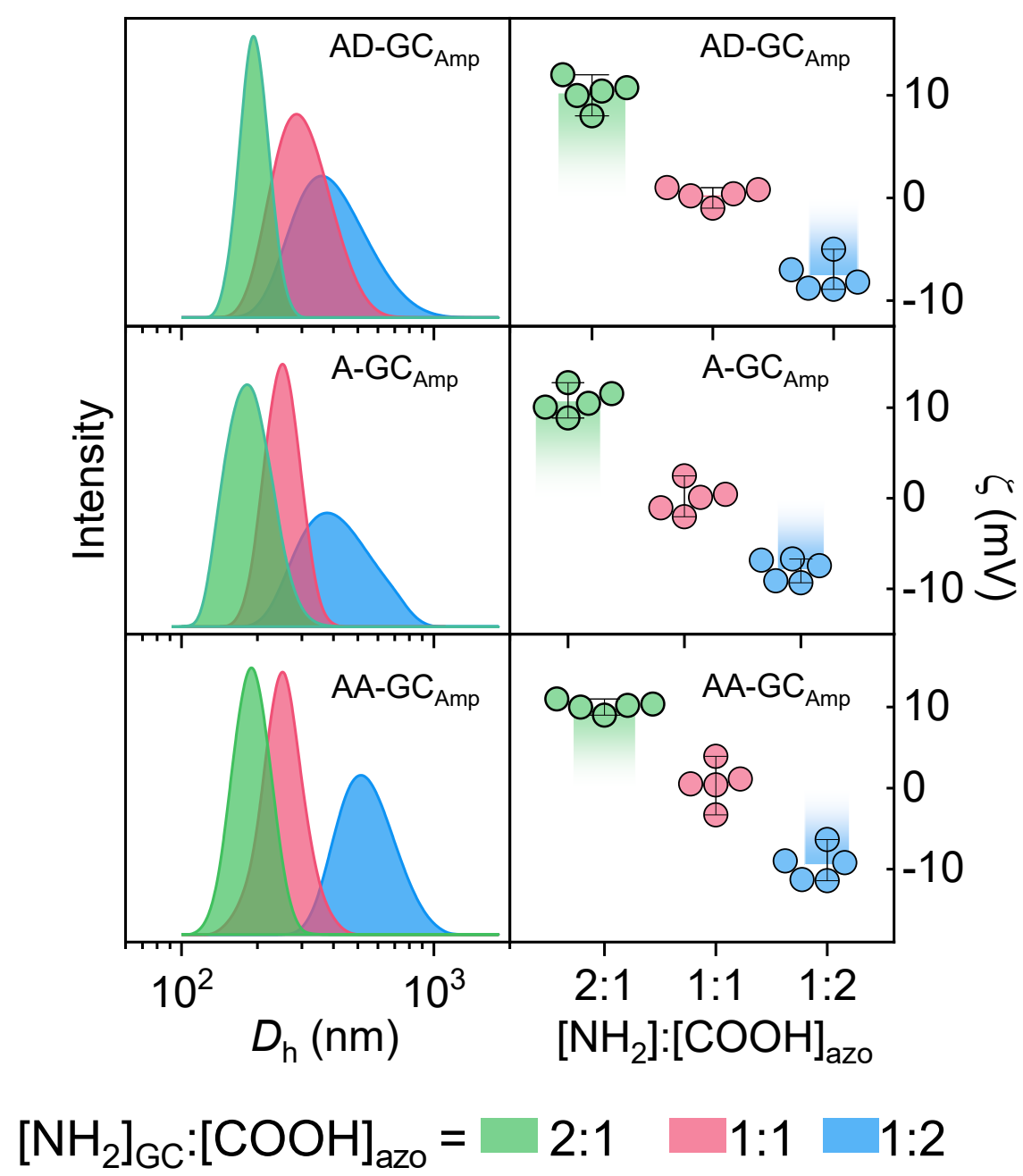

Figure S12. Hydrodynamic diameter distribution and zeta potential of **azo-GC$_{Amp}$** NPs at varying $[NH_2]_{GC}$:$[COOH]_{azo}$ ratios in PBS (pH 7.4).

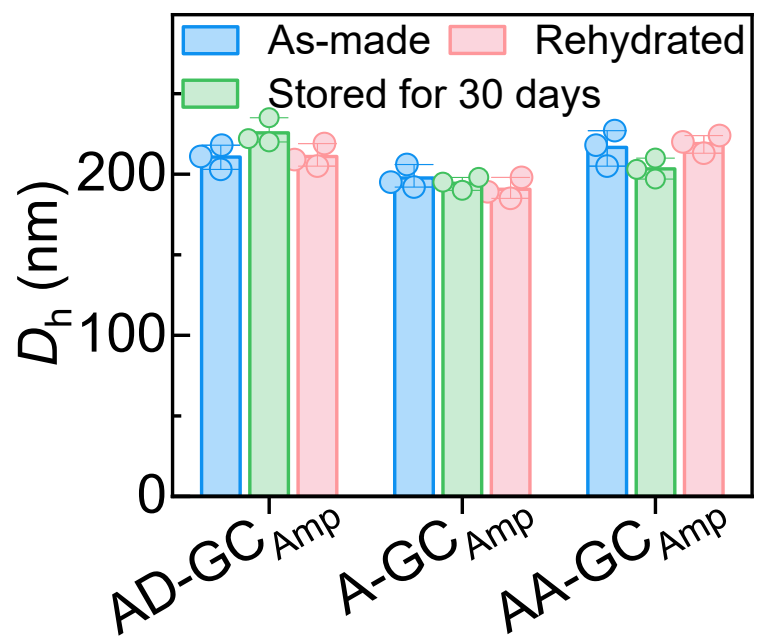

Figure S13. Hydrodynamic diameters of the as-made **azo-GC$_{Amp}$** NPs at an $[NH_2]_{GC}$:$[COOH]_{azo}$ ratio of 2:1, samples stored at 4 ^{o}C for 30 d, and rehydrated samples after lyophilization.

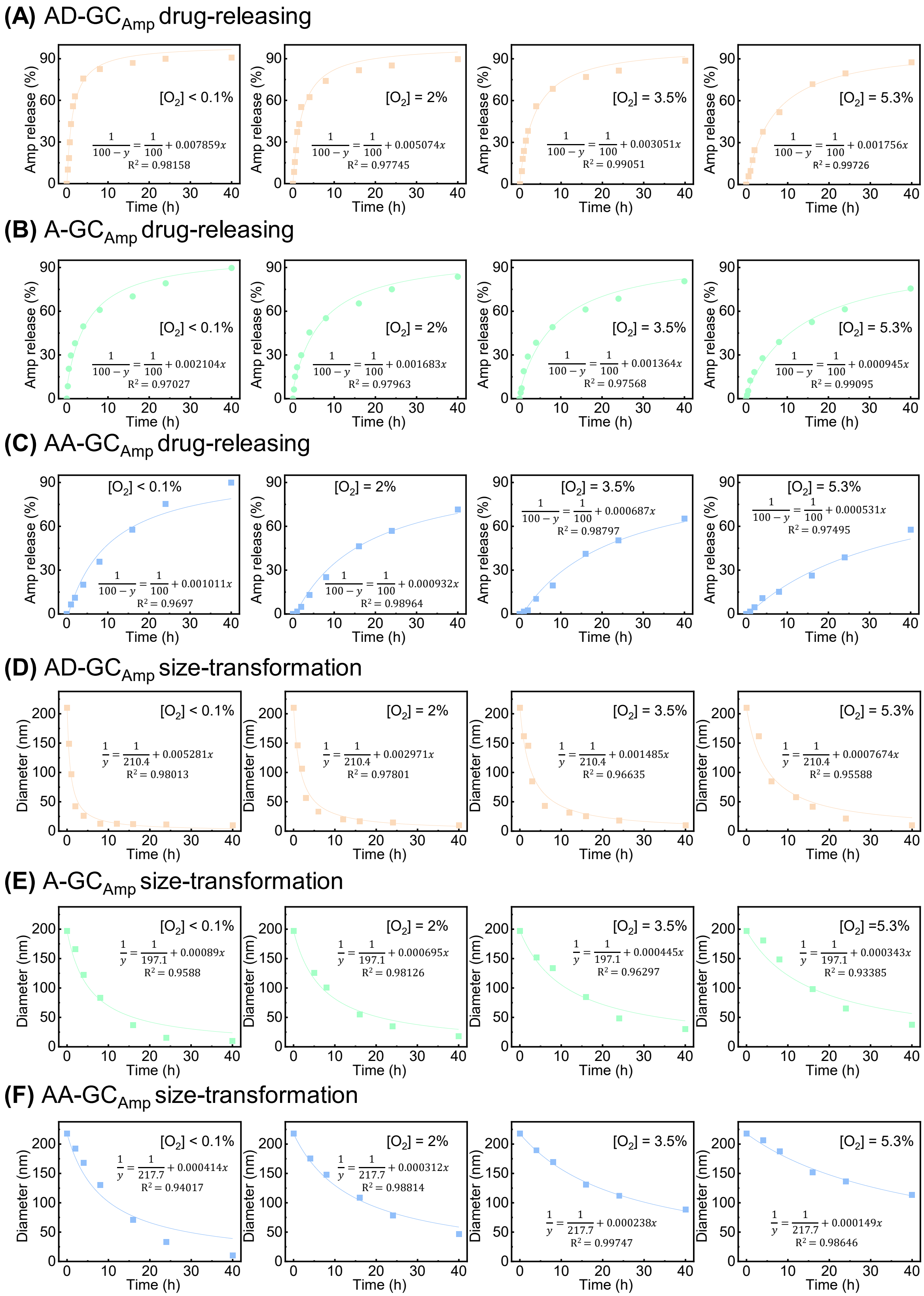

(A) AD-GC$_{Amp}$ drug-releasing
Amp release (%)
Time (h)
$[O_2] < 0.1\%$
$\frac{1}{100-y} = \frac{1}{100} + 0.007859x$
$R^2 = 0.98158$
$[O_2] = 2\%$
$\frac{1}{100-y} = \frac{1}{100} + 0.005074x$
$R^2 = 0.97745$
$[O_2] = 3.5\%$
$\frac{1}{100-y} = \frac{1}{100} + 0.003051x$
$R^2 = 0.99051$
$[O_2] = 5.3\%$
$\frac{1}{100-y} = \frac{1}{100} + 0.001756x$
$R^2 = 0.99726$
(B) A-GC$_{Amp}$ drug-releasing
$[O_2] < 0.1\%$
$\frac{1}{100-y} = \frac{1}{100} + 0.002104x$
$R^2 = 0.97027$
$[O_2] = 2\%$
$\frac{1}{100-y} = \frac{1}{100} + 0.001683x$
$R^2 = 0.97963$
$[O_2] = 3.5\%$
$\frac{1}{100-y} = \frac{1}{100} + 0.001364x$
$R^2 = 0.97568$
$[O_2] = 5.3\%$
$\frac{1}{100-y} = \frac{1}{100} + 0.000945x$
$R^2 = 0.99095$
(C) AA-GC$_{Amp}$ drug-releasing
$[O_2] < 0.1\%$
$\frac{1}{100-y} = \frac{1}{100} + 0.001011x$
$R^2 = 0.9697$
$[O_2] = 2\%$
$\frac{1}{100-y} = \frac{1}{100} + 0.000932x$
$R^2 = 0.98964$
$[O_2] = 3.5\%$
$\frac{1}{100-y} = \frac{1}{100} + 0.000687x$
$R^2 = 0.98797$
$[O_2] = 5.3\%$
$\frac{1}{100-y} = \frac{1}{100} + 0.000531x$
$R^2 = 0.97495$
(D) AD-GC$_{Amp}$ size-transformation
Diameter (nm)
$[O_2] < 0.1\%$
$\frac{1}{y} = \frac{1}{210.4} + 0.005281x$
$R^2 = 0.98013$
$[O_2] = 2\%$
$\frac{1}{y} = \frac{1}{210.4} + 0.002971x$
$R^2 = 0.97801$
$[O_2] = 3.5\%$
$\frac{1}{y} = \frac{1}{210.4} + 0.001485x$
$R^2 = 0.96635$
$[O_2] = 5.3\%$
$\frac{1}{y} = \frac{1}{210.4} + 0.0007674x$
$R^2 = 0.95588$
(E) A-GC$_{Amp}$ size-transformation
$[O_2] < 0.1\%$
$\frac{1}{y} = \frac{1}{197.1} + 0.00089x$
$R^2 = 0.9588$
$[O_2] = 2\%$
$\frac{1}{y} = \frac{1}{197.1} + 0.000695x$
$R^2 = 0.98126$
$[O_2] = 3.5\%$
$\frac{1}{y} = \frac{1}{197.1} + 0.000445x$
$R^2 = 0.96297$
$[O_2] =5.3\%$
$\frac{1}{y} = \frac{1}{197.1} + 0.000343x$
$R^2 = 0.93385$
(F) AA-GC$_{Amp}$ size-transformation
$[O_2] < 0.1\%$
$\frac{1}{y} = \frac{1}{217.7} + 0.000414x$
$R^2 = 0.94017$
$[O_2] = 2\%$
$\frac{1}{y} = \frac{1}{217.7} + 0.000312x$
$R^2 = 0.98814$
$[O_2] = 3.5\%$
$\frac{1}{y} = \frac{1}{217.7} + 0.000238x$
$R^2 = 0.99747$
$[O_2] = 5.3\%$
$\frac{1}{y} = \frac{1}{217.7} + 0.000149x$
$R^2 = 0.98646$

Figure S14. The fitting of (A-C) drug-releasing and (D-F) size-transformation process of **azo-GC$_{\mathbf{Amp}}$** NPs in the presence of azoreductase/NADPH at different oxygen conditions. The equations of $1/y = 1/y_0 + kt$ and $1/(100-y) = 1/100 + kt$ were used to fit the kinetics curves of size-transformation and drug releasing, respectively. The fittings were performed via OriginPro 2024, where k, t, and y_0 represent the rate constant, time, and initial y-axis value, respectively.

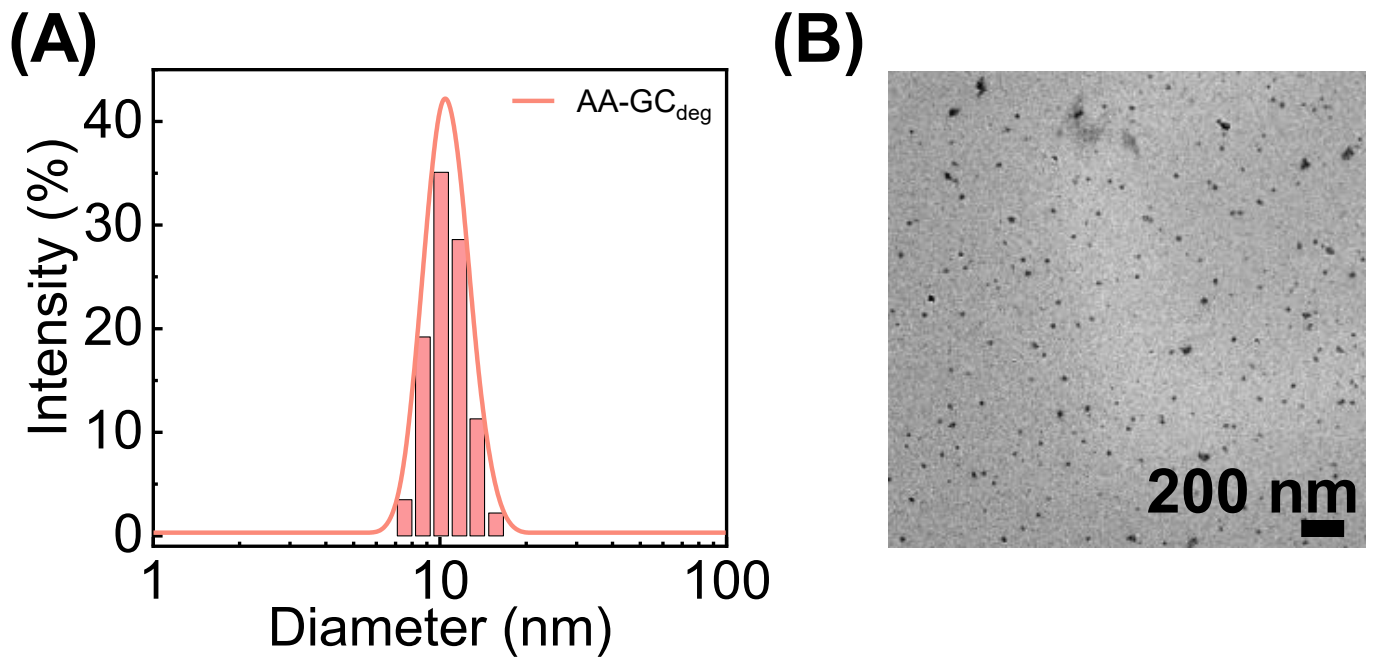

Figure S15. (A) DLS and (B) TEM of AA-GC$_{deg}$ prepared from AA-GC.

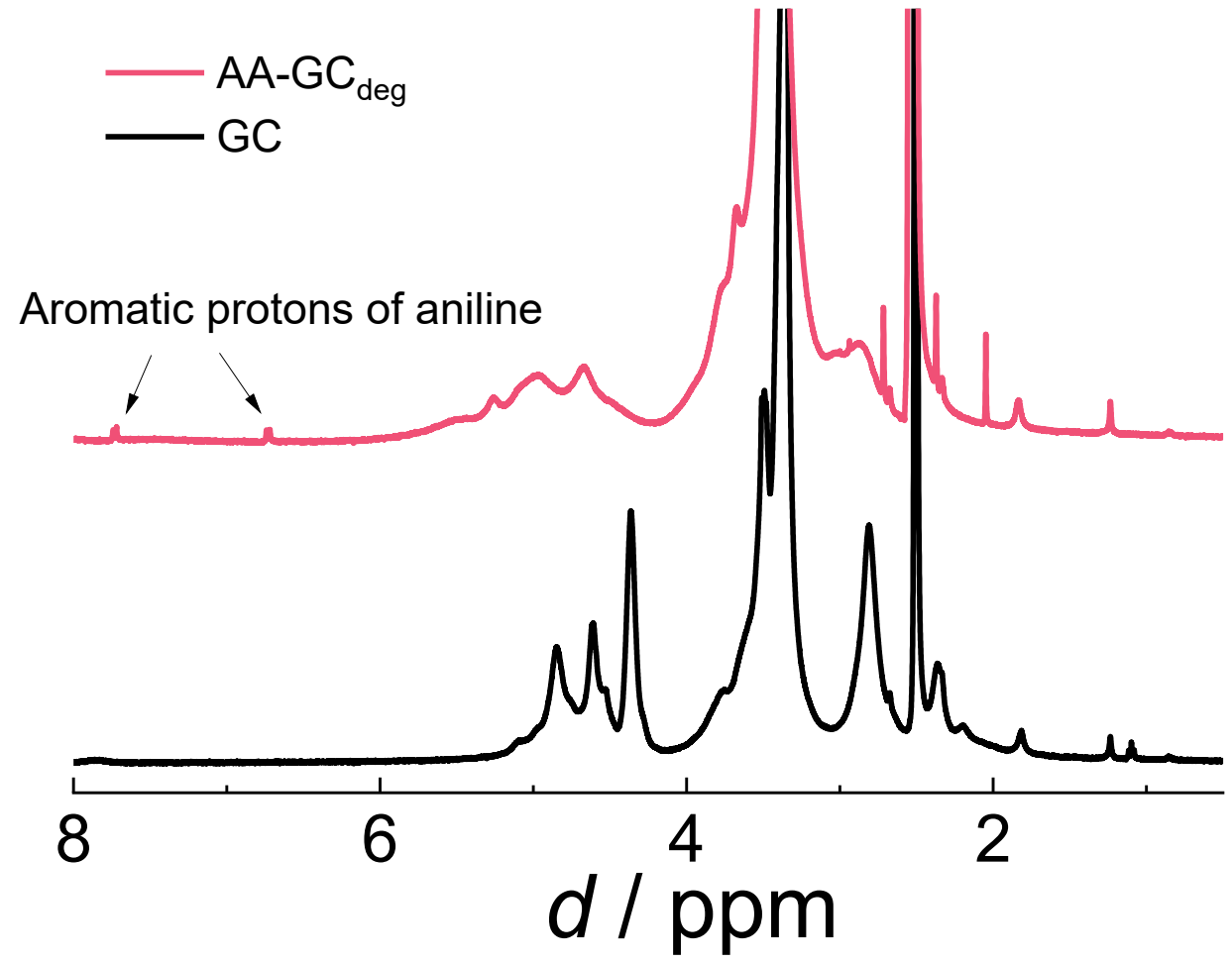

Figure S16. ^{1}H NMR spectra of GC and AA-GC$_{deg}$ in DMSO-d_6.

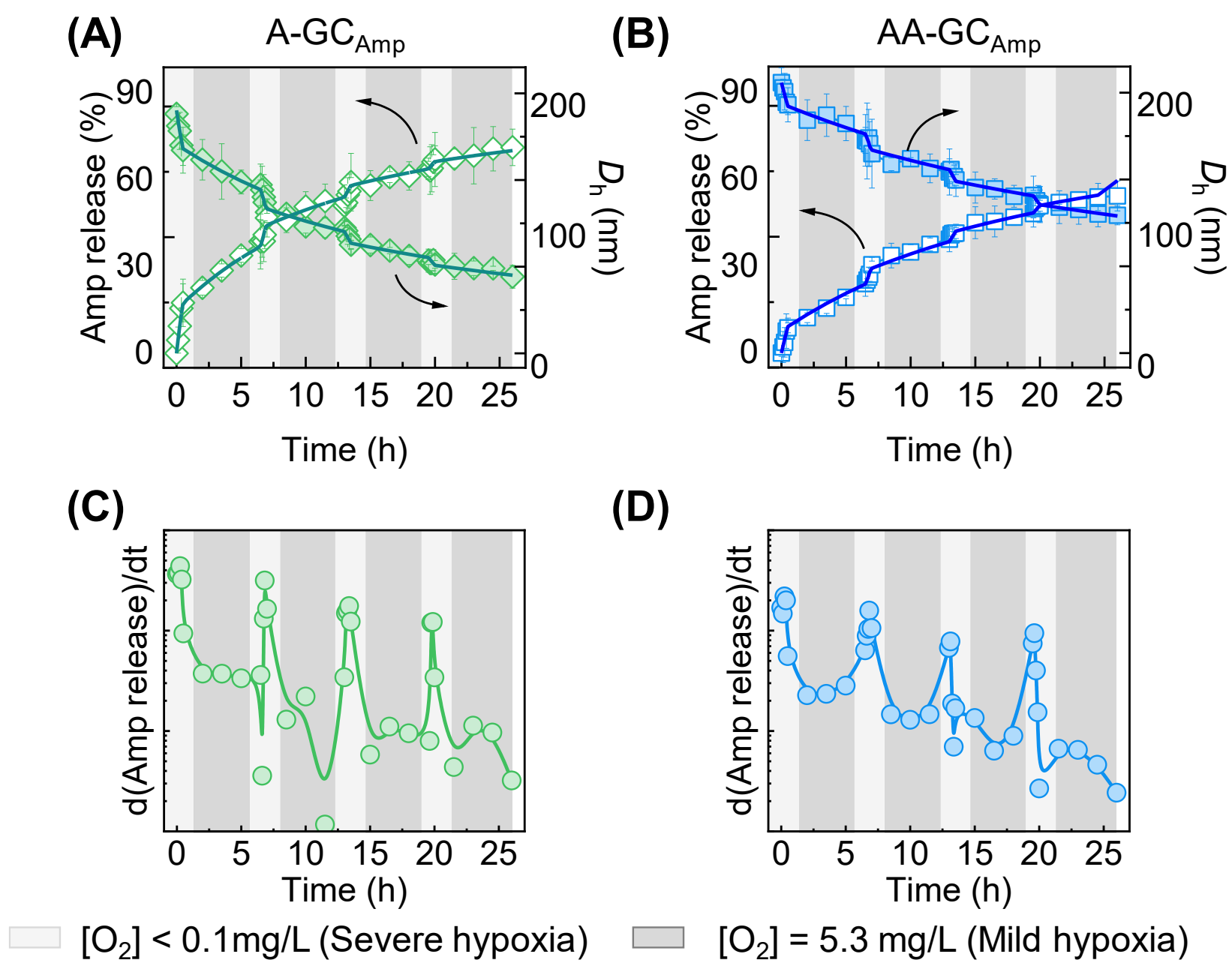

Figure S17. Switchable antibiotic release profiles of **azo-GC$_{Amp}$** NPs under different hypoxic conditions. (A, B) Time-dependent profiles of Amp release percentage and hydrodynamic diameters of **A-GC$_{Amp}$** (A) and **AA-GC$_{Amp}$** NPs (B) after incubation with azoreductase/NADPH under four cycles of alternating oxygen concentration (<0.1 and 5.3 mg/L). (C, D) Differential curves of Amp release profiles corresponding to A-GC$_{Amp}$ (C) and AA-GC$_{Amp}$ NPs (D), respectively.

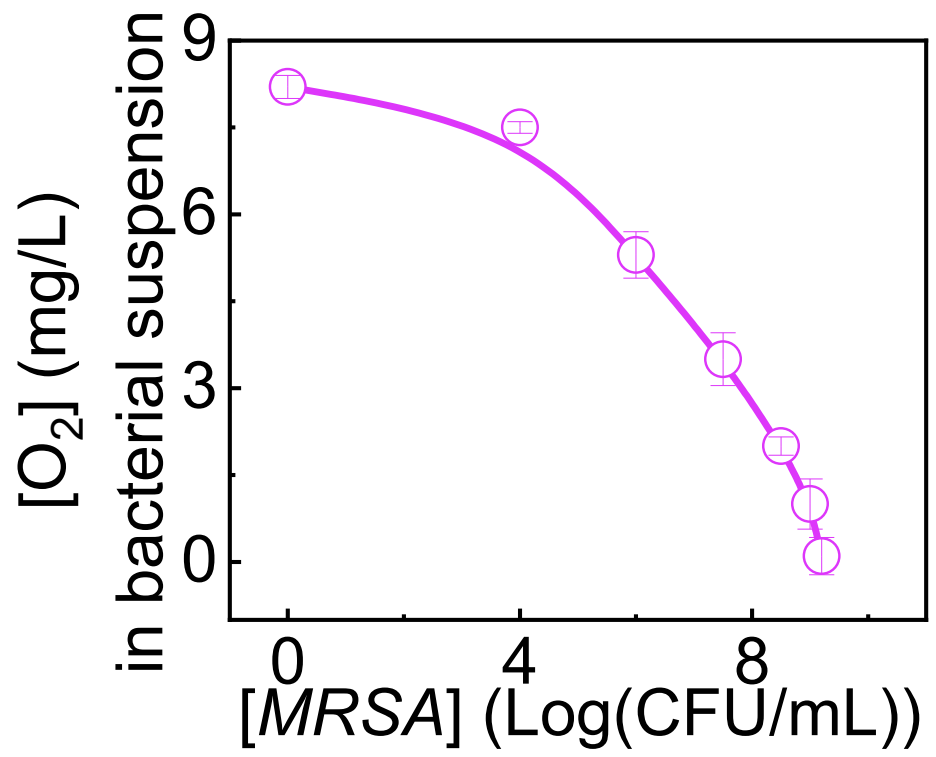

Figure S18. Oxygen concentration in the growth media as a function of MRSA bacterial concentration.

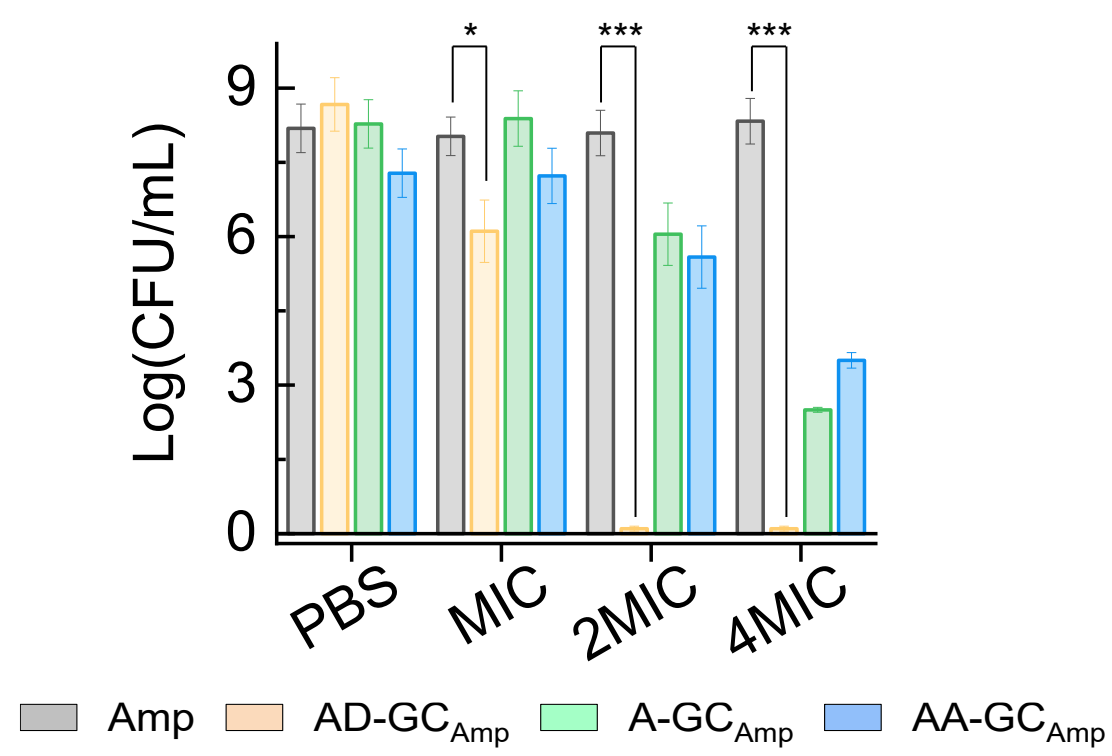

Figure S19. Histograms showing the viable bacteria counts of *E. coli* biofilms after 24 h treatment with **azo-GC$_{Amp}$** NPs at different concentrations (based on the MIC of Amp). PBS- and Amp-treated samples were used as controls.

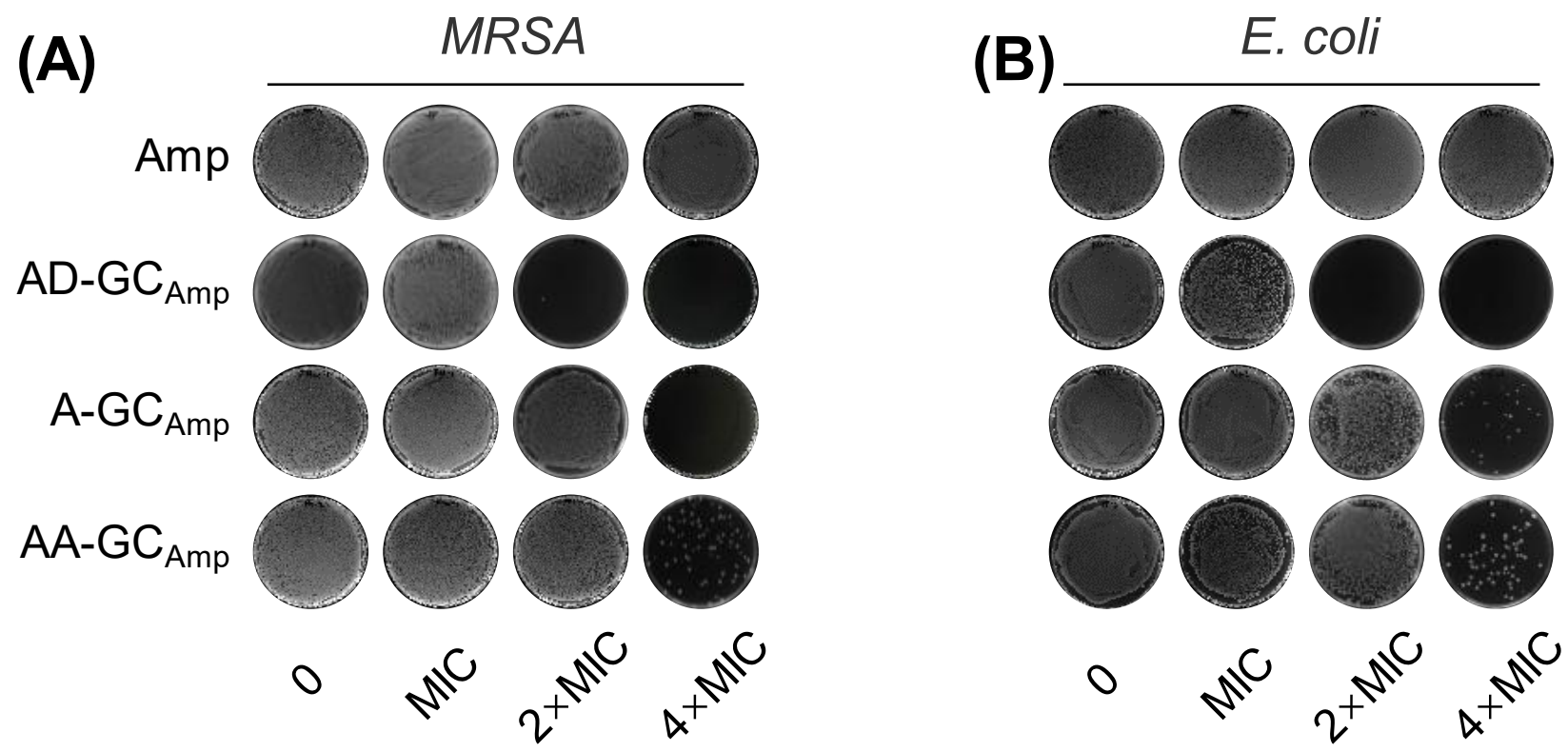

Figure S20. Photographs showing viable bacteria of (A) *MRSA and* (B) *E. coli* biofilms after the treatment with Amp and **azo-GC$_{Amp}$** NPs at various Amp concentrations.

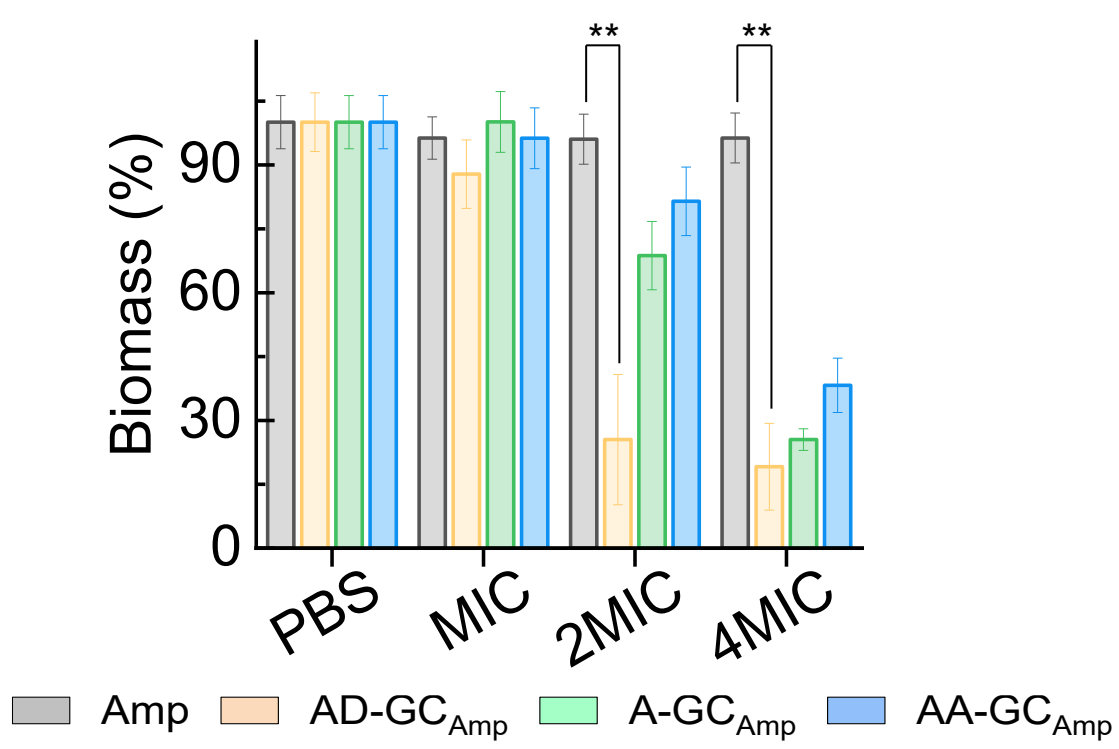

Figure S21. Histograms showing the biomass of *E. coli* biofilms after 24 h treatment with **azo-GC$_{Amp}$** NPs at different concentrations (based on the MIC of Amp). PBS- and Amp-treated samples were used as controls.

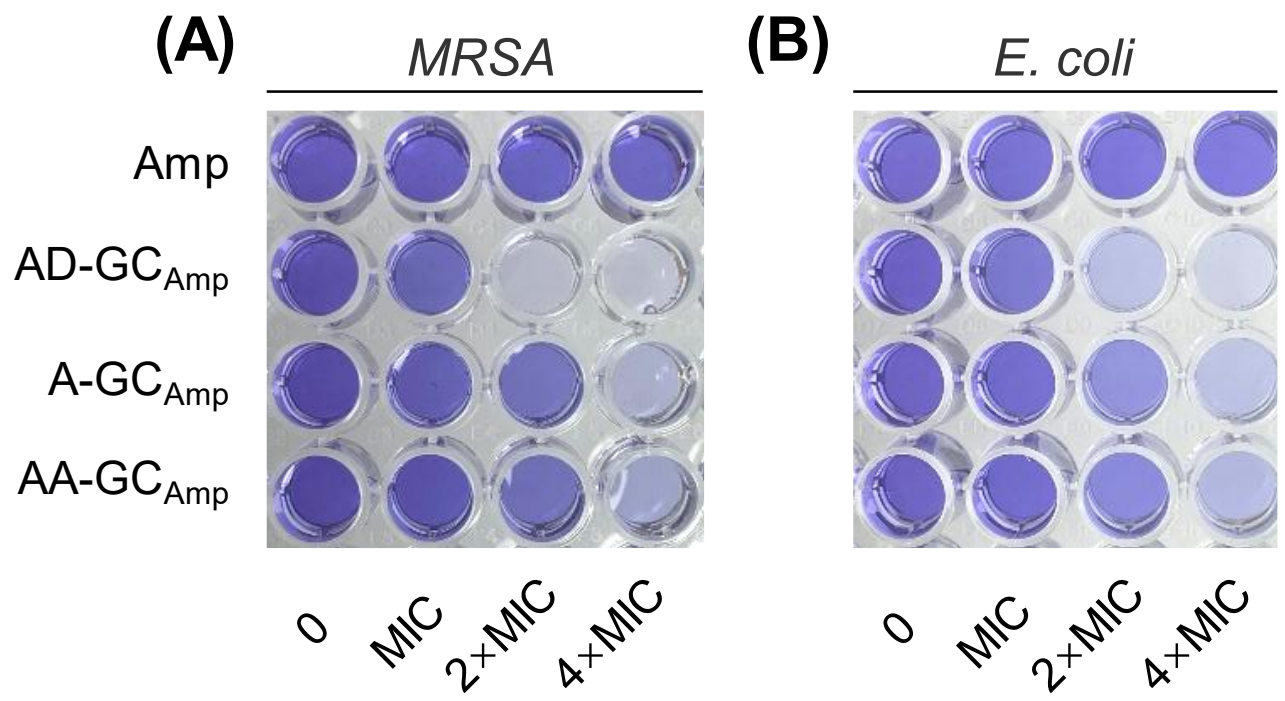

Figure S22. Photographs showing biomass of (A) *MRSA and* (B) *E. coli* biofilms after the treatment with Amp and **azo-GC$_{\mathbf{Amp}}$** NPs at various Amp concentrations.

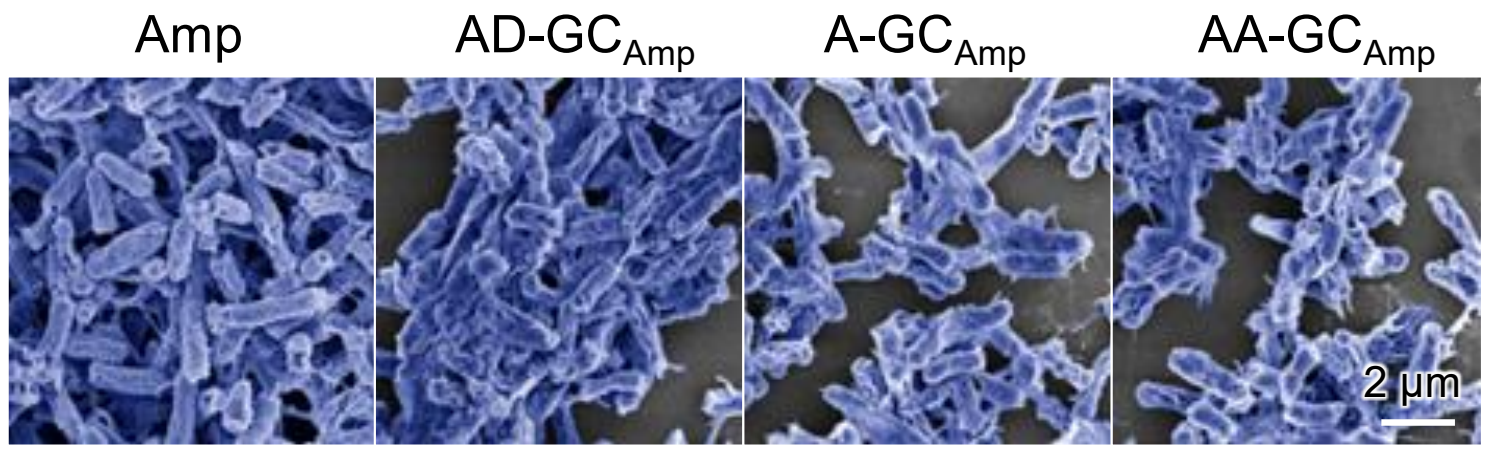

Figure S23. SEM images of *E. coli* biofilms after 24 h incubation with **azo-GC$_{Amp}$** NPs at 4×MIC (based on the MIC of Amp). Amp-treated samples were used as controls.

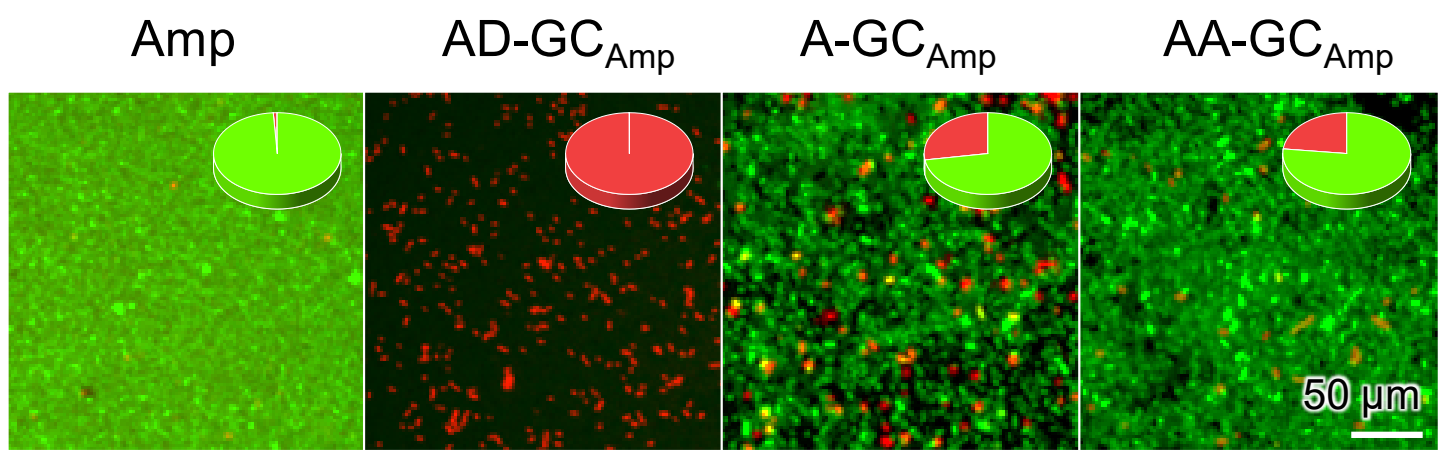

Figure S24. CLSM images of *E. coli* biofilms after 24 h incubation with **azo-GC$_{Amp}$** NPs at 4×MIC (based on the MIC of Amp). Amp-treated samples were used as controls.

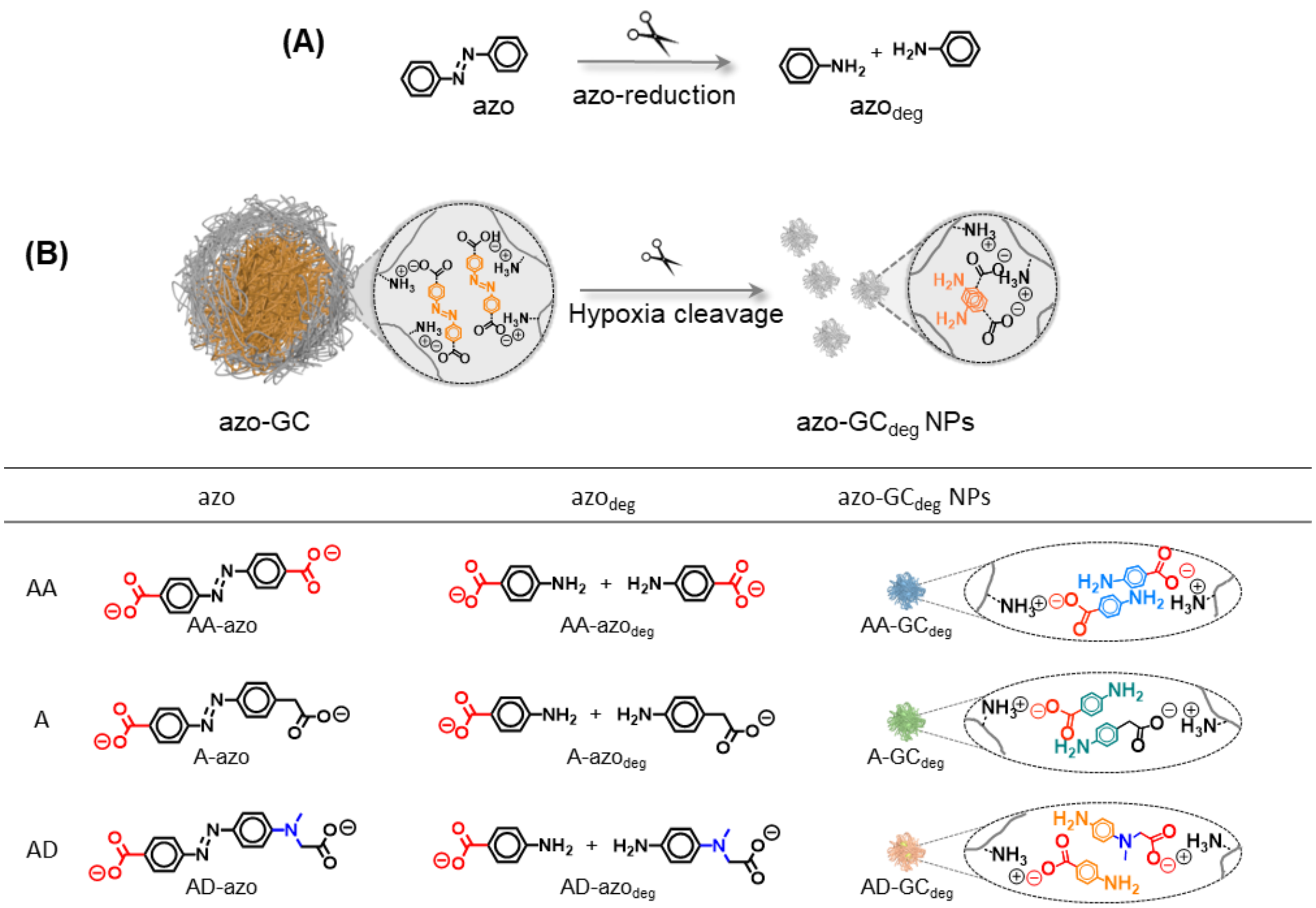

Figure S25. Schematic representation of the preparation of (A) azo$_{deg}$ and (B) degradation products of azo-based crosslinkers (**AD-azo$_{deg}$**, **A-azo$_{deg}$**, **AA-azo$_{deg}$**) and **azo-GC** NPs (**AD-GC$_{deg}$**, **A-GC$_{deg}$**, **AA-GC$_{deg}$**).

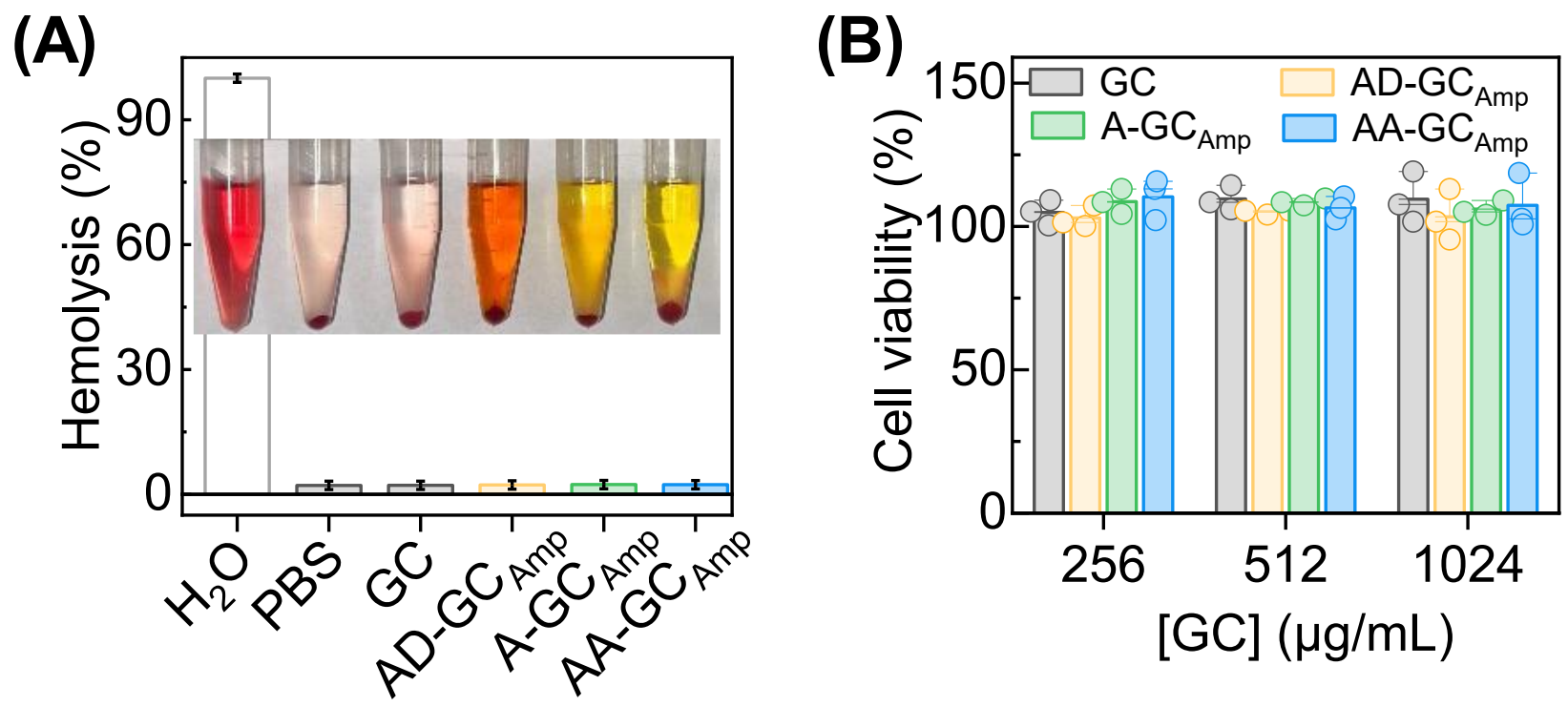

Figure S26. Biocompatibility assessment of **GC** polymer and **azo-GC$_{Amp}$** NPs. (A) Percent hemolysis of RBCs at a fixed **GC** concentration (1024 μg/mL) and (B) 3T3 cell viability at varying **GC** concentrations.

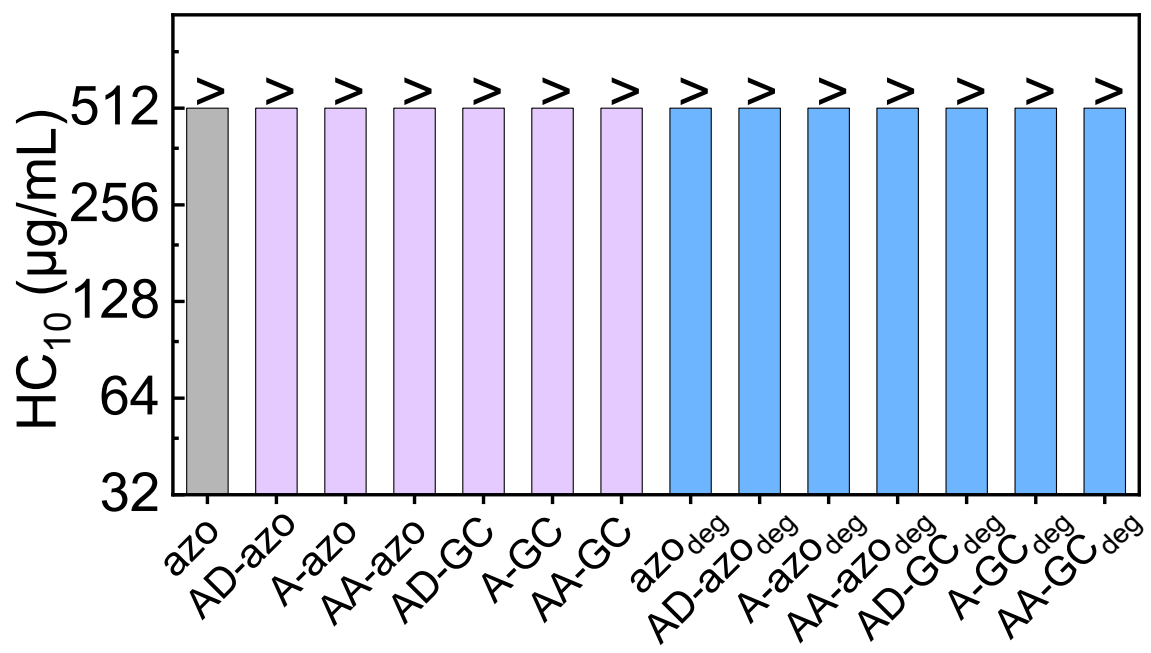

Figure S27. HC_{10} concentrations for different groups, derived from the hemolysis percentages of RBCs induced by azo-crosslinkers, **azo-GC** NPs, and their degradation products.

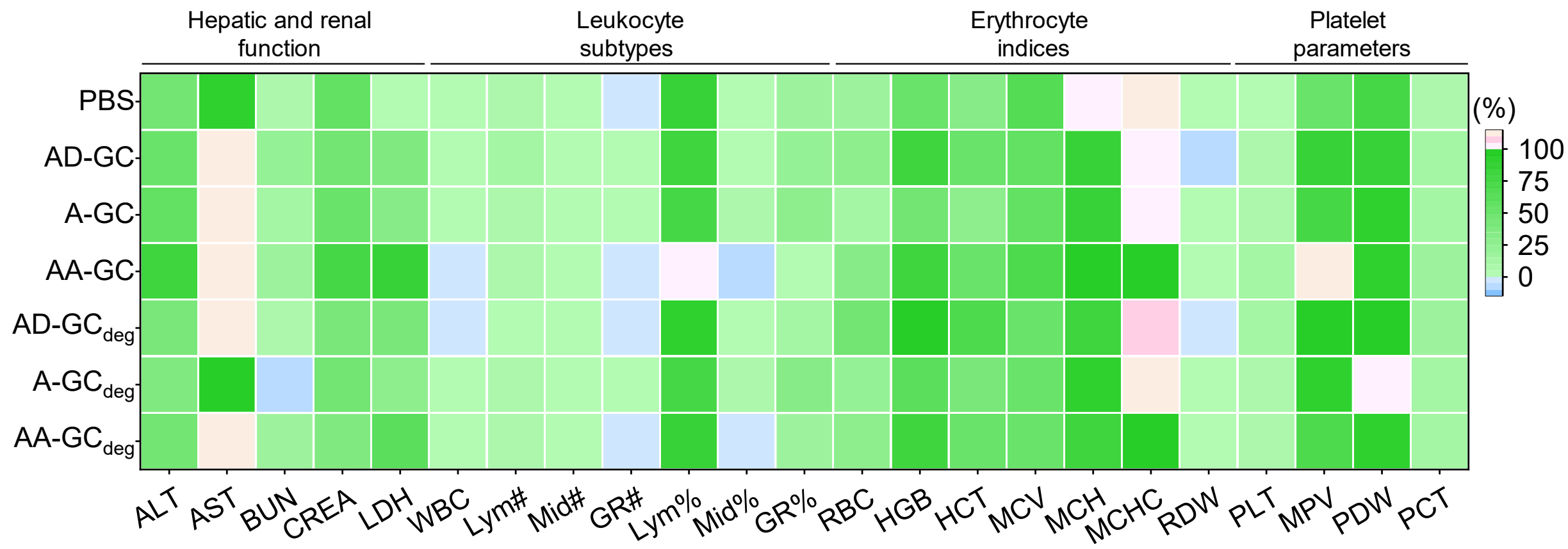

Figure S28. Routine blood and biochemical analysis results showing systemic toxicity at 24 h post-intravenous injection of **azo-GC NPs** and their degradation products.

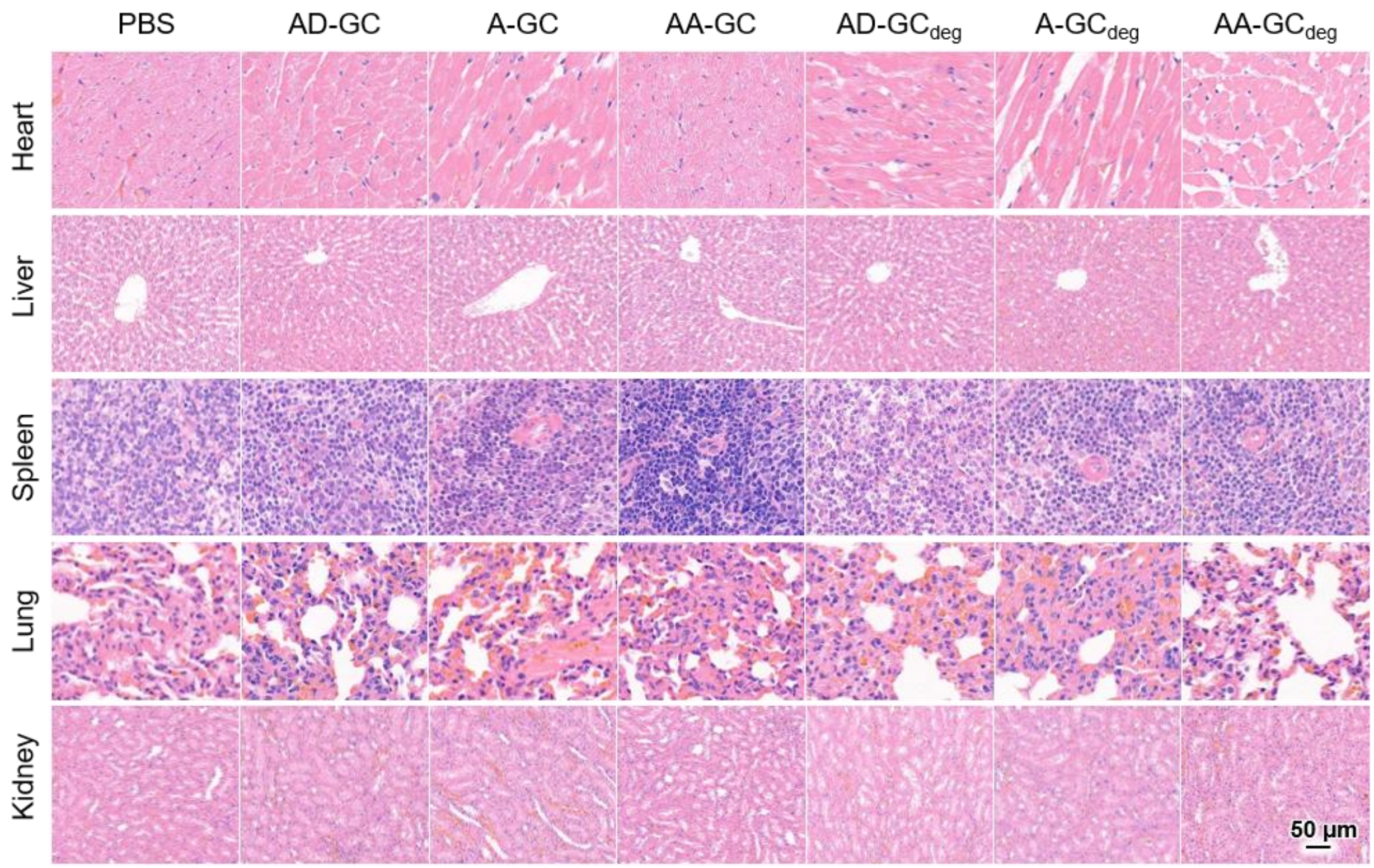

Figure S29. H&E-stained images of the hearts, liver, spleen, lungs, and kidneys showing histological biocompatibility at 7 days post-intravenous injection of **azo-GC** NPs and their degradation products.

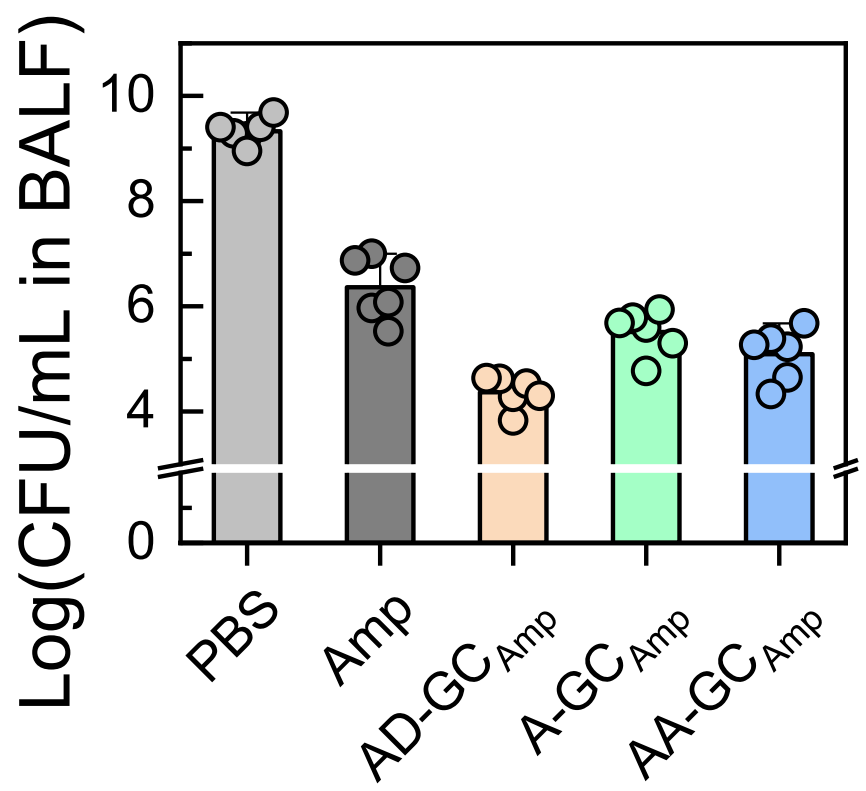

Figure S30. Quantification of viable bacteria in BALF after treatment with different therapies in an acute pulmonary infection model.

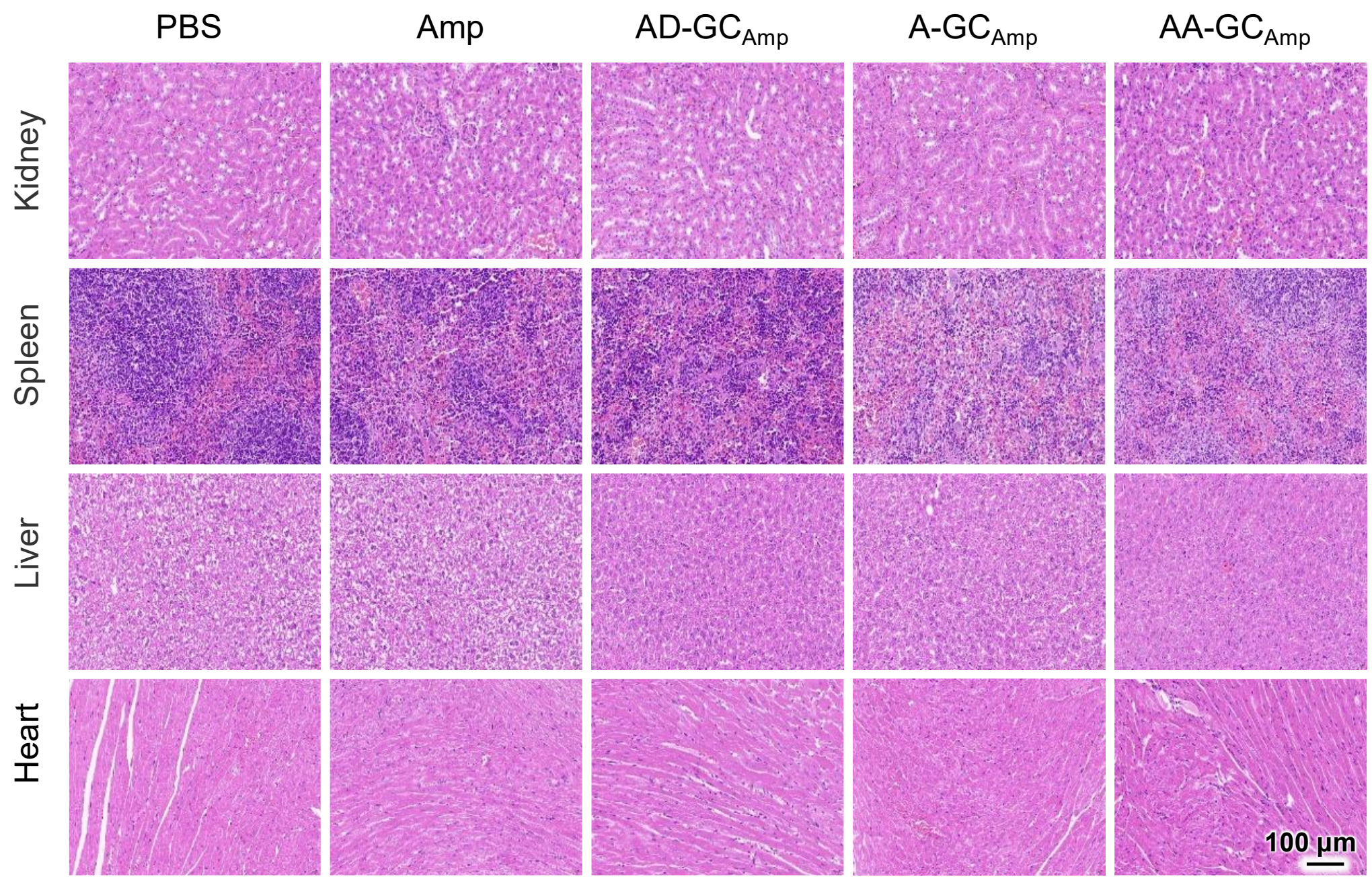

Figure S31. H&E-stained images of the kidneys, spleen, liver, and hearts showing tissue toxicity after different treatments at the experimental endpoint in an acute pulmonary infection model.

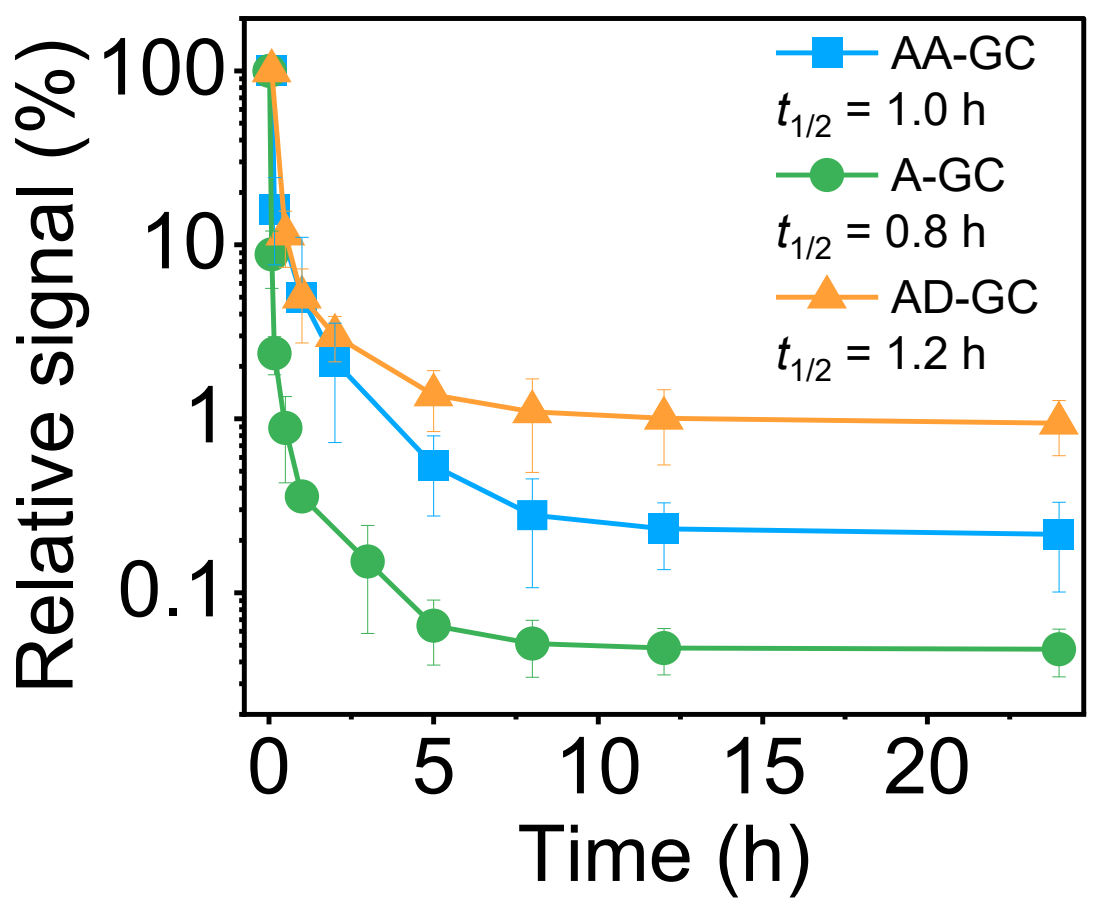

Figure S32. Blood circulation profiles of **AA-GC$_{Cy5.5}$**, **A-GC$_{Cy5.5}$**, and **AD-GC$_{Cy5.5}$** nanocarriers in healthy mice mice following *i.v.* injection at 15 mg/kg (n = 3). Plasma fluorescence intensity of each nanocarrier was measured at multiple time points post intravenous injection (0.09, 0.5, 1, 2, 4, 8, 12, and 24 h). The elimination half-lives ($t_{1/2}$) were determined as 1.0 h, 0.8 h, and 1.2 h for **AA-GC**, **A-GC**, and **AD-GC**, respectively. All three formulations displayed comparable circulation profiles.

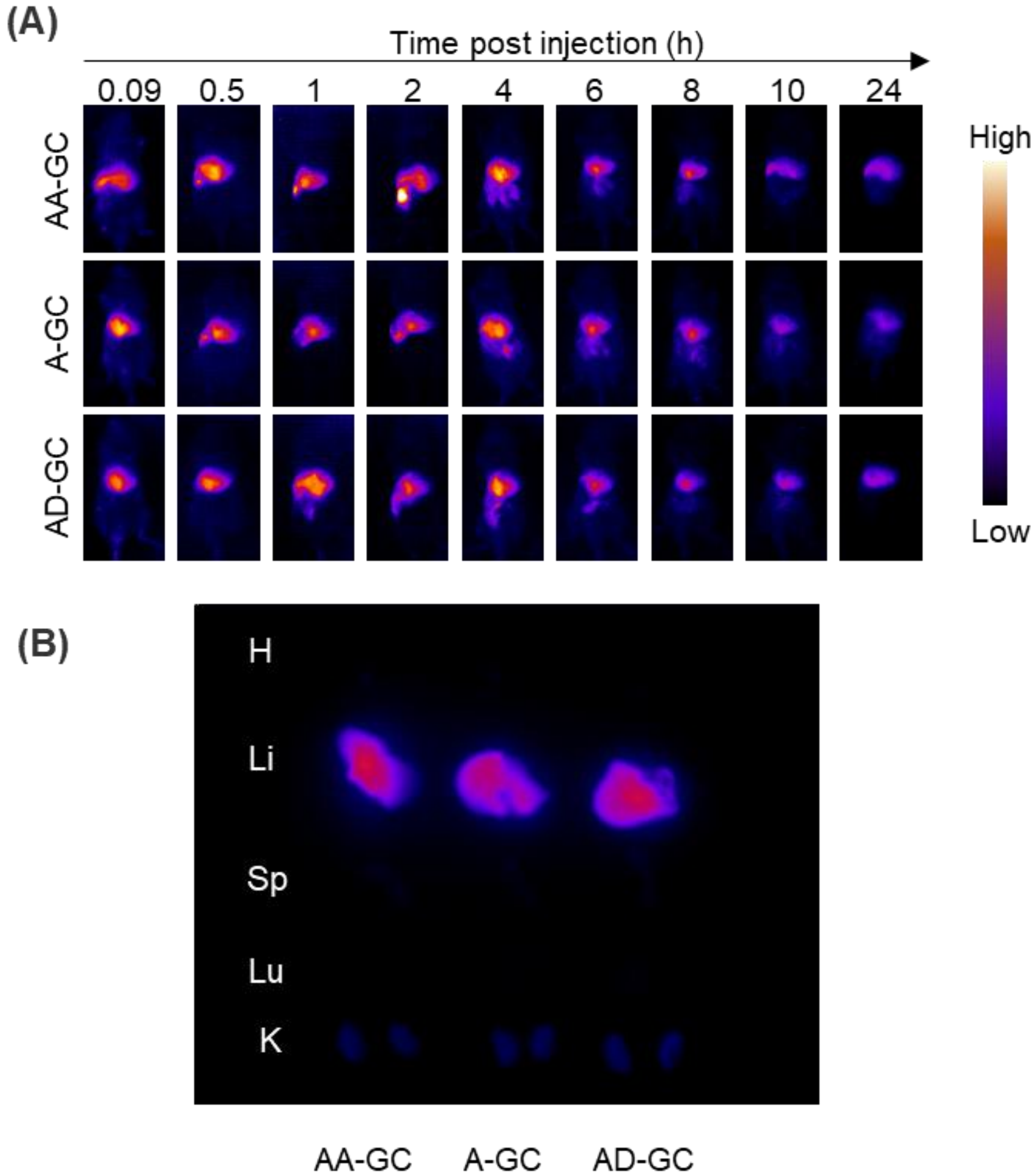

Figure S33. (A) Whole-body NIR fluorescence imaging of mice injected with **AA-GC**, **A-GC**, and **AD-GC** NPs. Representative images captured at 0.09, 1, 4, 8, 12, and 24 h post-injection show similar systemic clearance behaviors among the three nanocarriers. Fluorescence intensity gradually decreased in the bloodstream over time, consistent with elimination half-life measurements. (B) Ex vivo organ biodistribution at 24 h post-injection at 15 mg/kg. Fluorescence images of major organs ((H: heart; Li: liver Sp: spleen; Lu: lung; K: kidney) demonstrate that all three nanocarriers predominantly accumulate in the liver and spleen, consistent with reticuloendothelial system uptake. No significant differences in organ distribution were observed among the carriers.

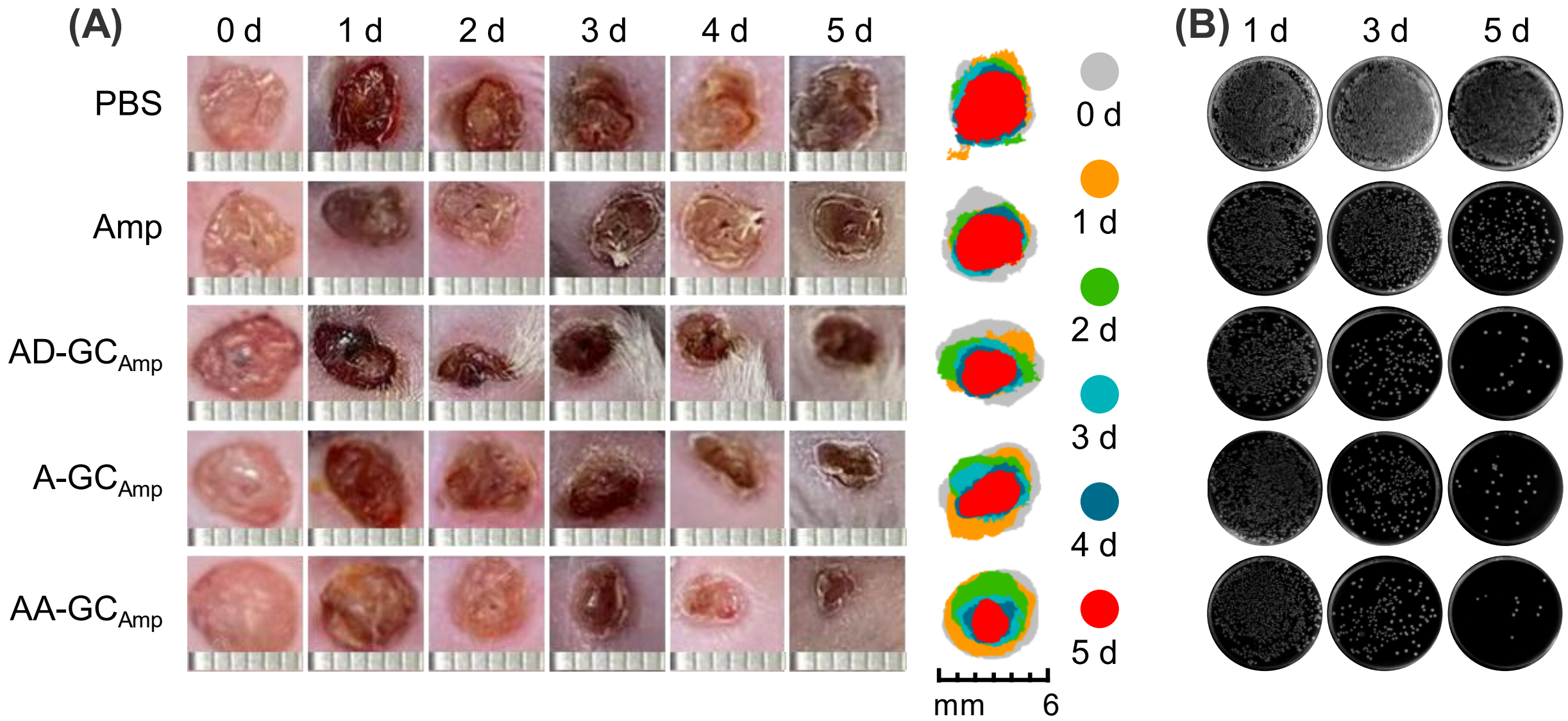

Figure S34. In vivo treatment assessment of an *E. coli*-infected wound model. (A) Representative wound images following various treatments over time and (B) photographs of bacterial agar plates for viable *E. coli* load quantification.

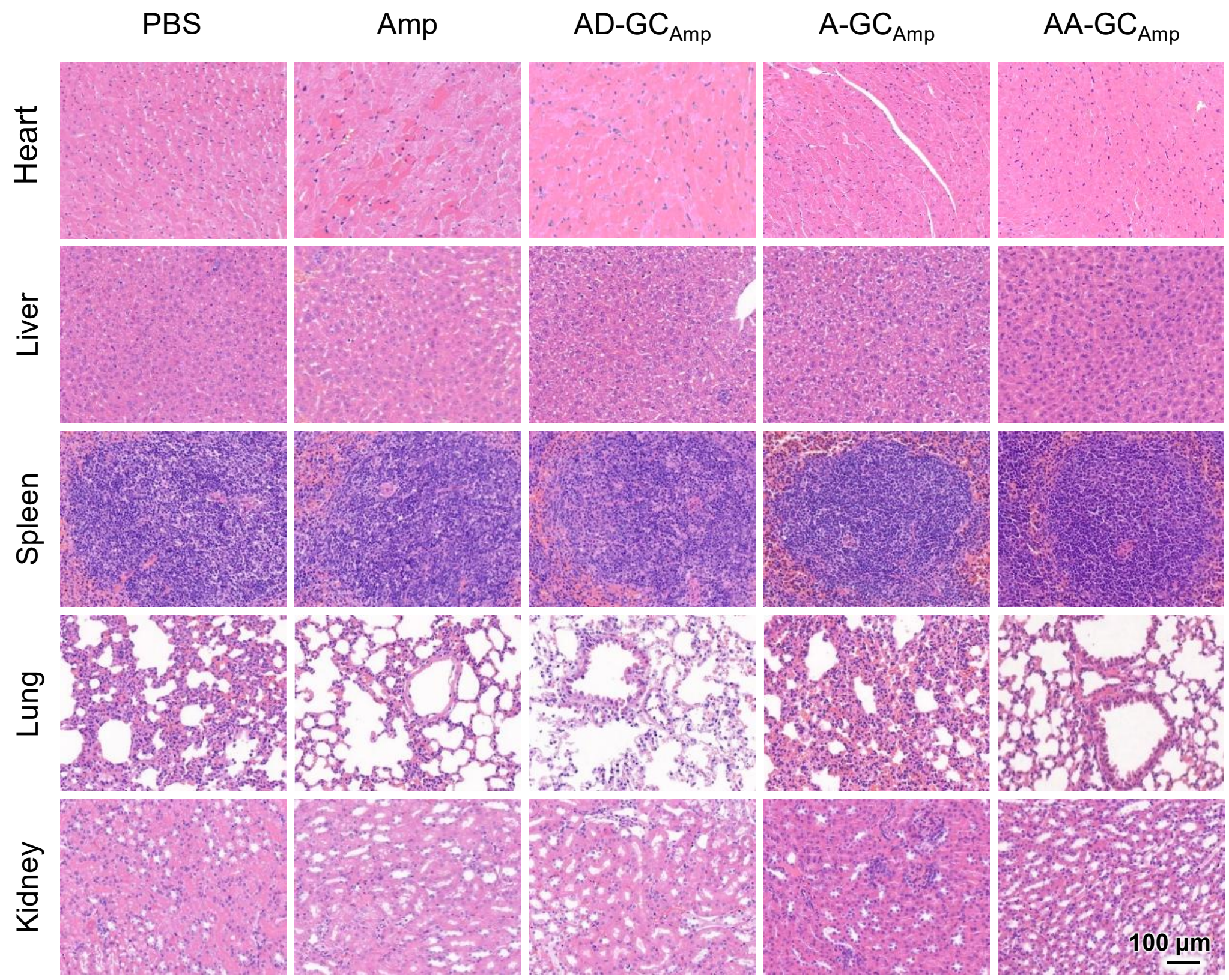

Figure S35. H&E-stained images of the hearts, liver, spleen, lungs, and kidneys showing long-term toxicity after different treatments at the experimental endpoint in an *E. coli* infection wound model.

Table S1. Calculated half-life ($t_{1/2}$) values of azo-based crosslinkers and **azo-GC$_{Amp}$** NPs in the presence of azoreductase/NADPH under hypoxic conditions ([O_2] <0.1 mg/mL).

**Samples**	**AD-type**		**A-type**		**AA-type**	
	**AD-azo**	**AD-GC$_{Amp}$**	**A-azo**	**A-GC$_{Amp}$**	**AA-azo**	**AA-GC$_{Amp}$**
**$t_{1/2}$ (h)**[a]	1.1	1.2	5.1	5.5	10.7	11.3

[a]$t_{1/2}$: The half-life of azoreduction in azo-based crosslinkers and **azo-GC$_{Amp}$** NPs, defined as the time required for the absorption intensity to decrease by 50%, calculated from Figure S11.

Table S2. Characterization of **azo-GC$_{Amp}$** NPs.

Samples	$D_{h,\, As\ made}$ (nm)[a]	Drug loading (As made, %)[b]	Drug loading (Rehydrated, %)[b]	Drug loading (Stored for 30 d, %)[b]	Encapsulation efficiency (%)[c]
**AD-GC$_{Amp}$**	210 ± 7.5	4.8 ± 0.2	4.7 ± 0.1	4.7 ± 0.3	31.7 ± 3.3
**A-GC$_{Amp}$**	197 ± 7.4	4.5 ± 0.3	4.6 ± 0.2	4.6 ± 0.1	29.6 ± 3.6
**AA-GC$_{Amp}$**	205 ± 1.1	3.8 ± 0.2	3.6 ± 0.3	3.7 ± 0.4	24.8 ± 2.5

[a]D_h: Hydrodynamic diameter, determined by DLS. [b]Drug loading content: Calculated using the equation: Drug loading capacity (%) = $\frac{Mass\ of\ loaded\ Amp}{Mass\ of\ NPs} \times 100\%$. [c]Encapsulation efficiency: Calculated using the equation: Encapsulation efficiency (%) = $\frac{Mass\ of\ loaed\ Amp}{Mass\ of\ fed\ Amp} \times 100\%$.

Table S3. Calculated half-lives ($t_{1/2}$) for the drug-release and size transformation processes of **azo-GC$_{Amp}$** NPs in the presence of azoreductase/NADPH under varying oxygen levels.

[O_2]	AD-GC$_{Amp}$		A-GC$_{Amp}$		AA-GC$_{Amp}$	
(mg/L)	$t_{1/2, drug}$ (h)[a]	$t_{1/2, size}$ (h)[b]	$t_{1/2, drug}$ (h)[a]	$t_{1/2, size}$ (h)[b]	$t_{1/2, drug}$ (h)[a]	$t_{1/2, size}$ (h)[b]
5.3	6.5	6.2	14.0	14.8	32.7	30.9
3.5	3.7	3.2	9.1	11.4	22.3	19.3
2	2.2	1.6	7.1	7.3	15.0	14.7
< 0.1	1.4	0.9	5.3	5.7	11.0	11.1

[a]$t_{1/2}$, Half-life of the hypoxia-induced drug release process, calculated from Figures S14A-S14C.
[b]$t_{1/2}$, Half-life of the hypoxia-induced size transformation process, calculated from Figure S14D-S14F.

Table S4. Biological activities of Amp antibiotics and **azo-GC$_{Amp}$** NPs under different oxygen levels.

	$[O_2]$ [mg/L][a]	MIC[b] [μg/mL]		HC_{10}[e] [μg/mL]	IC_{50}[f] [μg/mL]
		MRSA[c]	*EC*[d]		
Amp	< 0.1	32	8	>1024	>1024
	2	32	8		
	3.5	32	8		
	5.3	32	8		
	8	32	8		
AD-GC$_{Amp}$	< 0.1	32	8	>1024	>1024
	2	32	8		
	3.5	64	16		
	5.3	256	32		
	8	512	128		
A-GC$_{Amp}$	< 0.1	32	8	>1024	>1024
	2	32	8		
	3.5	128	32		
	5.3	256	64		
	8	512	128		
AA-GC$_{Amp}$	< 0.1	64	16	>1024	>1024
	2	64	16		
	3.5	128	64		
	5.3	512	128		
	8	512	128		

[a]$[O_2]$: Dissolved oxygen concentration. [b]MIC: Minimum inhibitory concentration; the lowest concentration required to inhibit >90% bacterial growth. [c]MRSA: Methicillin-resistant *Staphylococcus aureus,* ATCC 43300. [d]*EC*: *Escherichia coli*, ATCC 25922. [e]HC_{10}: The lowest concentration required to cause 10% lysis of RBCs. [f]IC_{50}: The concentration that causes 50% inhibition of 3T3 proliferation.

Data S1. (separate file). Optimized Cartesian coordinates of all molecular structures used in the DFT calculations.
